# Inhibition of cathepsin L ameliorates inflammation through the A20/NF-κB pathway in endotoxin-induced acute lung injury

**DOI:** 10.1016/j.isci.2024.111024

**Published:** 2024-10-04

**Authors:** Shiyi Yang, Kaijun Chen, Jinkang Yu, Zhangchu Jin, Min Zhang, Zhouyang Li, Yang Yu, Nanxia Xuan, Baoping Tian, Na Li, Zhengtong Mao, Wenbing Wang, Tianpeng Chen, Yinfang Wu, Yun Zhao, Min Zhang, Xia Fei, Songmin Ying, Wen Li, Fugui Yan, Xingxian Zhang, Gensheng Zhang, Huahao Shen, Zhihua Chen

**Affiliations:** 1 Key Laboratory of Respiratory Disease of Zhejiang Province, Department of Respiratory and Critical Care Medicine, Second Affiliated Hospital of Zhejiang University School of Medicine, Hangzhou, Zhejiang 310009, China; 2 Department of Critical Care Medicine, Second Affiliated Hospital of Zhejiang University School of Medicine, Hangzhou, Zhejiang 310009, China; 3 College of Pharmaceutical Sciences, Zhejiang University of Technology, Hangzhou, Zhejiang 310014, China; 4 International Institutes of Medicine, The Fourth Affiliated Hospital of Zhejiang University School of Medicine, Yiwu 322000, China; 5 Department of Pharmacology & Department of Respiratory and Critical Care Medicine of the Second Affiliated Hospital, Zhejiang University School of Medicine, Key Laboratory of Respiratory Disease of Zhejiang Province, Hangzhou 310009, China; 6 State Key Lab of Respiratory Disease, Key Cite of National Clinical Research Center for Respiratory Disease, Guangzhou 510120, China

**Keywords:** Biological sciences, Molecular biology, Molecular interaction

## Abstract

Acute lung injury (ALI)/acute respiratory distress syndrome (ARDS) is a severe inflammatory condition that remains refractory; however, its molecular mechanisms are largely unknown. Previous studies have shown numerous compounds containing 4-indolyl-2-aminopyrimidine that display strong anti-inflammatory properties. In our research, we identified that a 4-Indole-2-Arylaminopyrimidine derivative named “IAAP” suppressed lipopolysaccharide (LPS)-induced inflammation. Immunoprecipitation and liquid chromatography-tandem mass spectrometry (LC-MS/MS) identified that IAAP interacts with a lysosomal cysteine protease, cathepsin L (CTSL), and restrains its activity. The nuclear factor kappa B (NF-κB) family plays a central role in controlling innate immunity. Canonical NF-κB activation, such as stimulation with lipopolysaccharide (LPS), typically involves the degradation of A20. We observed that IAAP suppression of CTSL prevented the LPS-induced degradation of A20, thereby ameliorating NF-κB activation. This study identifies CTSL as a crucial regulator of A20/NF-κB signaling and suggests IAAP as a potential lead compound for developing drugs to treat ALI/ARDS.

## Highlights

• A small molecule IAAP ameliorates LPS-induced inflammatory responses and ALI/ARDS
• IAAP interacts with and suppresses the bioactivity of CTSL
• CTSL is upregulated in BALF of patients with ARDS and the murine model of ALI
• CTSL degrades the deubiquitinase A20 and promotes the activation of NF-κB signaling

## Graphical abstract

**Figure:**
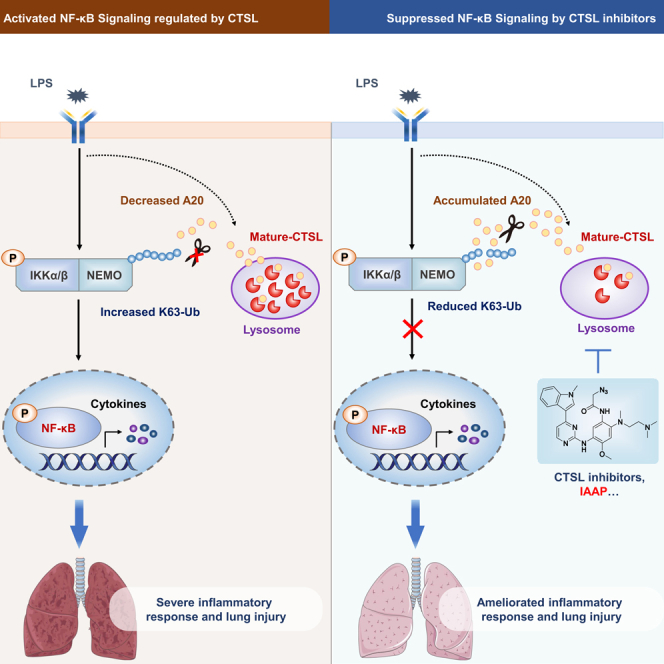

## Introduction

Acute lung injury (ALI)/acute respiratory distress syndrome (ARDS) is a fatal and undertreated condition characterized by increased pulmonary vascular permeability, massive inflammation, pulmonary edema, and refractory hypoxia. Clinically, it manifests as hypoxemia with bilateral opacities on chest radiography, in addition to reduced lung compliance and enhanced physiological dead space.^1^^,^^2^ Studies have shown that the incidence of ALI/ARDS is 70/100 000 and 59/100 000 annually, respectively, with a case fatality rate of approximately 40%.^3^ Furthermore, the coronavirus of 2019 (COVID-19) pandemic has resulted in 6 million deaths worldwide, primarily due to complications from severe acute respiratory syndrome coronavirus 2 (SARS-CoV-2)-associated ARDS, supporting the urgent need to better understand its molecular pathogenesis.^4^ To date, there are relatively few treatments available for ALI/ARDS, and the cornerstone of management is mechanical ventilation; drug therapy may differ depending on the individual patient and the inciting cause.^5^^,^^6^ The proposed therapeutic drugs include common chemical drugs, antibiotics, and biomacromolecular drugs composed of proteins, polypeptides, and genetic materials (such as RNA).^7^^,^^8^^,^^9^^,^^10^ However, no drug has been shown to significantly reduce the mortality rate in ALI/ARDS in clinical trials.^5^^,^^11^^,^^12^ Therefore, it is of great significance to identify new intervention targets for the clinical prevention and treatment of ALI/ARDS.

ALI/ARDS can be caused by various pulmonary insults (such as pneumonia and aspiration) or non-pulmonary causes (such as sepsis, pancreatitis, and trauma).^13^ Lipopolysaccharide (LPS) is the primary component of the outer membrane of Gram-negative bacteria and is capable of inducing a wide range of infections, such as severe pneumonia and septicemia.^14^ Signaling also activates the downstream nuclear factor kappa-B (NF-κB) and augments inflammatory mediators.^15^ Hence, LPS has emerged as a clinically relevant model for ALI/ARDS.^16^ The NF-κB family is a key regulator of innate and adaptive immune responses.^17^ In ALI/ARDS, LPS activates canonical NF-κB activation, which typically involves K63-polyubiquitinated NF-κB essential modulator (NEMO) and subsequent phosphorylation of IκBα.^18^^,^^19^

The lysosome is an active metabolic site in cells and the primary organelle for the decomposition and recycling of various biological macromolecules. Lysosomes are involved in a variety of life processes, including gene regulation, signal transduction, energy metabolism, and immunity, and are at the center of complex networks that regulate the stability of the internal environment of cells and organisms.^20^^,^^21^^,^^22^^,^^23^ Cathepsins are a family of multifunctional proteases that are synthesized and transported as zymogens into lysosomes and are activated by cleavage in the acidic environment of the lysosome.^22^^,^^24^^,^^25^ Studies have shown that cathepsins are crucial for antigen presentation, inflammasome signaling, vascular remodeling, and protein cleavage processing.^26^^,^^27^^,^^28^ Cathepsin L (CTSL) plays an important role in regulating antigen proteolysis and processing the MHC-bound invariant chain (li).^29^^,^^30^ The p41 splice variant of MHC class II-associated Ii binds noncovalently to the active site of CTSL and protects mature-CTSL from degradation.^31^^,^^32^ Recent studies have shown that CTSL is highly expressed in the respiratory system, and CTSL inhibitors, such as SID 26681509 and E64d, reduce live viral infection of *ex vivo* lung tissues of both human donors and human ACE2-transgenic mice.^33^^,^^34^ Eosinophil-derived cathepsin L promotes pulmonary matrix destruction and emphysema by degrading the extracellular matrix.^35^ However, whether lysosomal CTSL is involved in the development of ALI/ARDS remains unclear.

In the present study, we explored the effects and mechanisms of IAAP on LPS-induced responses. Our findings revealed that LPS facilitated CTSL-mediated degradation of A20, thereby promoting the activation of NF-κB signaling, whereas IAAP suppresses LPS-induced inflammatory responses by targeting and inhibiting lysosomal CTSL.

## Results

### IAAP ameliorates lipopolysaccharide-induced inflammatory response *in vitro* and acute lung injury *in vivo*

**Figure 1:**
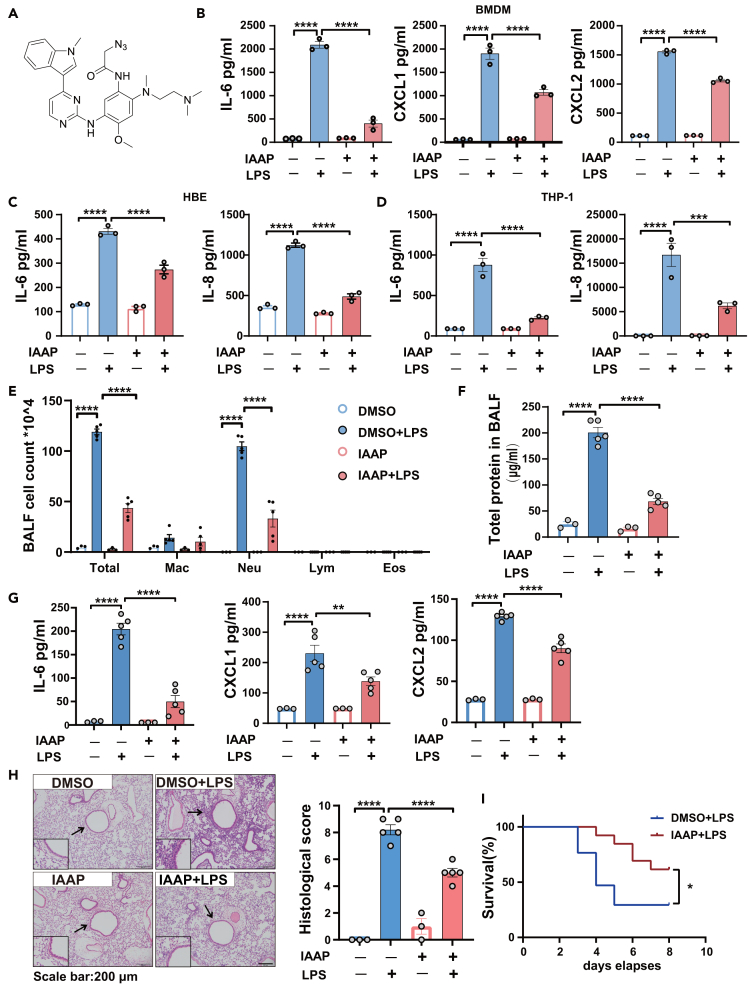
IAAP ameliorates LPS-induced inflammatory response *in vitro* and ALI *in vivo* (A) The chemical structure of IAAP. (B) BMDM were stimulated with LPS (100 ng/mL) or treated with IAAP (5 μM) for 6 h. Cells were then harvested to analyze secreted IL-6, CXCL1, and CXCL2 proteins in cell culture supernatants using ELISA, *n* = 3 each group. (C) HBE were stimulated with LPS (100 μg/mL), or treated with IAAP (5 μM) for 24 h. Cells were then harvested to analyze IL-6 and IL-8 proteins in cell culture supernatants using ELISA, *n* = 3 each group. (D) THP-1 were stimulated with LPS (100 ng/mL), or treated with IAAP (5 μM) for 6 h. Cells were then harvested for analyzing secreted IL-6 and IL-8 proteins in cell culture supernatants using ELISA, *n* = 3 each group. (E) Mice were treated by intraperitoneal injection with IAAP (20 mg/kg) 1h before pentobarbital injection and then with LPS (in 50 μL saline) at a dose of 5 mg/kg or NS though intratracheal administration. Mice were sacrificed 24 h after LPS administration for analysis. Total and differential cell counts in the BALF from mice: n (NS + DMSO) = 3, n (LPS + DMSO) = 5, n (IAAP) = 3, n (IAAP + LPS) = 5. (F) Total protein concentration in the BALF. (G) Expression of IL6, CXCL1, and CXCL2 in the BALF. (H) Representative images of lung tissue with HE staining 24 h post-LPS challenge and histological inflammatory scores. Scale bar: 200 μm. (I) For the survival assay, mice were intratracheally treated with LPS (20 mg/kg) on day 0. Mice received an intraperitoneal injection of IAAP (20 mg/kg) or PBS 1h before intratracheal instillation on day 0 and were injected with drugs twice on day 3. Survival rates of mice after the intratracheal administration of LPS with or without IAAP intraperitoneal injection for 24 h. n (DMSO + LPS) = 17, n (IAAP + LPS) = 18. DMSO was used as the vehicle control. All the quantitative data are presented as mean ± SEM and differences were identified using one-way ANOVA. The log rank (Mantel-Cox) test was used to analyze the survival rates. ∗*p* < 0.05, ∗∗*p* < 0.01, ∗∗∗*p* < 0.001, ∗∗∗∗*p* < 0.0001.

In previous studies, heterocyclic compounds containing indoles or pyrimidines were shown to have significant anti-inflammatory effects.^36^^,^^37^ We designed and synthesized IAAP using 4-indole-2-arylaminopyrimidine as the core skeleton.^38^ Based on classical drug design principles, minimum change principle, and bioisosterism, we improved the hydrophilicity and enhanced the biological activity of IAAP by replacing the fluorine atoms with various amino substituents at position four of the phenyl ring. The molecular structure of IAAP is shown in Figure 1A. We first examined the cell cytotoxicity of IAAP in BMDM, HBE, and THP-1 cells using MTT assays (Figure S1A), and IAAP showed no obvious cytotoxicity (cell survival rates ≥70%). To determine the optimal concentration of IAAP *in vitro* and *in vivo*, the protein levels of IL-6 induced by LPS were measured after treatment with different concentrations of IAAP in HBE, THP-1, and BMDM (Figures S1B–S1D), and the anti-inflammatory effects of different concentrations of IAAP in LPS-induced ALI in mice were evaluated (Figures S1E–S1G). Then the protein levels of IL-6, IL-8, CXCL1, and CXCL2 induced by LPS were significantly downregulated after treatment with 5 μM IAAP in BMDM, HBE, and THP-1 cells, respectively (Figures 1B–1D). In a murine model of intratracheal LPS-induced inflammation and ALI/ARDS, IAAP (20 mg/kg) led to a significant reduction in total cell counts, neutrophil numbers, and protein concentrations in the bronchoalveolar lavage fluid (BALF) compared to LPS-treated mice (Figures 1E and 1F). This protective effect was also confirmed by the reduced levels of chemokines and the pro-inflammatory cytokines CXCL1, CXCL2, and IL6 in the BALF (Figure 1G). Histopathological examination revealed that IAAP significantly reduced the severity of lung inflammation and damage (Figure 1H). As expected, the mice treated with IAAP exhibited significantly higher survival rates than the control group after LPS administration (Figure 1I). These results indicate that IAAP ameliorated the LPS-induced inflammatory response *in vitro* and ALI *in vivo*.

### IAAP impairs lysosomal degradation activity leading to the excessive accumulation of autolysosomes

**Figure 2:**
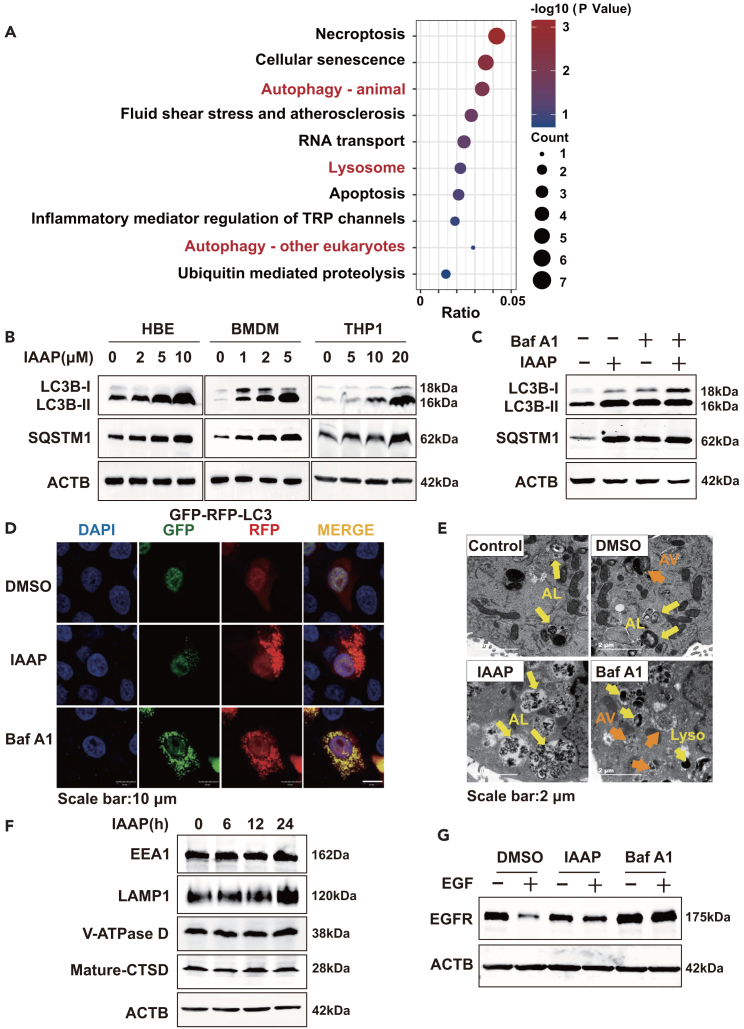
IAAP impairs lysosomal degradation activity leading to the excessive accumulation of autolysosomes (A) List of IAAP-related pathways. HBE cells were treated with biotin (10 μM), IAAP (10 μM), or IAAP-biotin (10 μM) for 24 h and then harvested for Co-IP. The resulting Co-IP protein solutions were used for protein identification by LC-MS/MS. LC-MS/MS results were used for KEGG pathway enrichment analysis. (B) HBE, BMDM, and THP-1 were treated with IAAP (5 μM) for 6 h, the levels of LC3B and SQSTM1 were examined using Western blotting. (C) HBE were treated with IAAP (5 μM) or Baf A1 (5 nM) for 6 h. Cellular LC3B and SQSTM1 levels were analyzed using Western blotting. (D) HBE expressing GFP-RFP-LC3B were treated with IAAP (5 μM) or Baf A1 (5 nM) for 6 h and imaged using confocal microscopy. Scale bar: 10 μm. (E) HBE was treated with IAAP (5 μM) or Baf A1 (5 nM) for 6 h, then the cells were imaged using electron microscopy. AV, autophagic vacuole/autophagosome; AL, autolysosome; Lyso, lysosome. Scale bar: 2 μm. (F) HBE were treated with IAAP (5 μM) for 6 h, and the levels of EEA1, LAMP1, Mature-CTSD, and V-ATPase D were examined using Western blotting. (G) Western blot analysis of EGFR at the indicated time points after EGF incubation in cells treated with IAAP (5 μM) or Baf A1 (5 nM) for 6 h. DMSO served as the vehicle control.

To investigate the underlying mechanisms of IAAP in LPS-induced inflammation, a biotin-labeled IAAP was designed and synthesized. We performed co-IP experiments with IAAP-biotin and used immunoprecipitation and liquid chromatography-tandem mass spectrometry (LC-MS/MS) to qualitatively analyze the protein molecules that interact with IAAP-biotin. LC-MS/MS revealed that IAAP-biotin could bind to 1913 proteins. After excluding 1744 proteins that bound non-specifically to biotin or Streptomycin C1, we screened 169 proteins that were specifically bound to IAAP. The full LC-MS/MS results are presented in Data S2. The identified proteins mapped to 169 genes. Kyoto Encyclopedia of Genes and Genomes (KEGG) pathway enrichment analysis of these genes revealed that IAAP primarily acts on steps in the autophagy-lysosome process (Figure 2A).

To determine whether IAAP regulates LPS-induced airway inflammation through autophagy-lysosome signaling, we evaluated the levels of autophagic proteins in different cell lines. When autophagy is activated, LC3B accumulates as an autophagy marker for autophagosome formation and the autophagy substrate SQSTM1 is preferentially degraded. The results revealed that LC3B expression was significantly increased in IAAP-treated cells (Figures 2B and S2A). In contrast, IAAP increased instead of decreased SQSTM1 protein levels in a dose-dependent manner, suggesting that autophagic degradation was inhibited by IAAP treatment (Figure 2B). Upon Bafilomycin A1 (Baf A1)-treatment, autophagic proteins reached levels similar to those of IAAP treatment alone, and there were few synergistic or additive effects between Baf A1 and IAAP (Figure 2C), suggesting that the accumulation of LC3B and SQSTM1 induced by IAAP resulted from decreased degradation of the autophagosome. Next, we monitored autophagy flux using the GFP-RFP-LC3B plasmid. Both red and green fluorescence was observed in Baf A1-treated cells, indicating autophagosome accumulation. Interestingly, IAAP treatment resulted in the accumulation of red dots with few punctate green fluorescence (Figure 2D). Transmission electron microscopy (TEM) revealed that IAAP triggered a significant accumulation of autolysosomes, displaying relatively large-sized single-membrane vesicles with visible cytoplasmic contents, whereas Baf A1 induced accumulation of autophagosomes (Figure 2E).

We assessed lysosomal biosynthesis and maturation. Lysosomes are progressively acidified by the action of vacuolar-type H + -ATPase (V-ATPase) as they mature from early endosomes. During maturation, pro-CTSD, an inactive propeptide, is cleaved into the mature enzyme in the acidic environment of lysosomes. Early Endosome Autoantigen-1 (EEA1), lysosomal-associated membrane protein 1 (LAMP1), and V-ATPase D were used to identify early endosomes, lysosomes, and V-ATPase, respectively. IAAP treatment led to a significant increase in LAMP1, but no change in the expression of EEA1, mature CTSD, or V-ATPase D (Figure 2F). Lyso-tracker confirmed that IAAP induced the accumulation of lysosomes (Figure S2B). We estimated the lysosomal degradation function based on the degradation of the epidermal growth factor receptor (EGFR).^39^ Western blotting analysis showed that EGFR degradation was suppressed in IAAP-treated cells (Figure 2G). Collectively, these results indicate that IAAP impairs lysosomal degradation activity without affecting lysosomal pH or fusion with autophagosomes, resulting in the excessive accumulation of autolysosomes.

### IAAP interacts with CTSB and cathepsin L to suppress their bioactivity, of which cathepsin L is critical for lysosomal function

**Figure 3:**
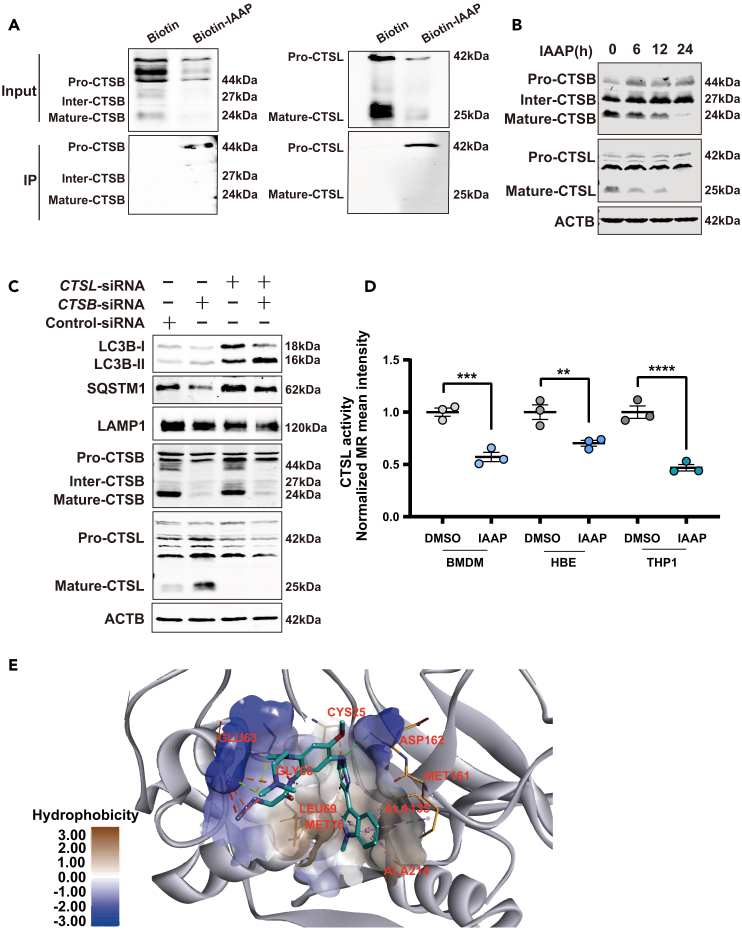
IAAP interacts with CTSB and CTSL to suppress their bioactivity, of which CTSL is critical for lysosomal function (A) HBE cells were treated with biotin (10 μM), or IAAP-biotin (10 μM) for 24 h, the cell lysates were immunoprecipitated with beads-streptavidin and subjected to immunoblotting. (B) HBE cells were treated with IAAP for 6 h, and the levels of CTSL and CTSB were examined using Western blotting. (C) HBE cells were transfected with control-, *CTSB*- or *CTSL*-siRNA for 24 h. The levels of LC3B, SQSTM1, LAMP1, CTSB, and CTSL were analyzed using Western blotting. (D) BMDM and THP-1 cells were treated with IAAP (5 μM) for 6 h, and HBE were treated with IAAP (5 μM) for 24 h. The CTSL activity in BMDM, HBE, and THP-1 cells was evaluated, *n* = 3 each group. (E) 3D schematic representation of IAAP bound to CTSL subsites. Best docking poses of IAAP at the active site of CTSL. The most relevant interacting residues were present in orange carbon polytubes, and ligands in blue carbon polytubes. DMSO was used as the vehicle control. All the quantitative data are presented as mean ± SEM and differences were identified using one-way ANOVA. ∗∗*p* < 0.01, ∗∗∗*p* < 0.001, and ∗∗∗∗*p* < 0.0001.

As shown previously (Figures 2C–2G), IAAP inhibited lysosomal protein degradation. Based on the LC-MS/MS results, we speculated that IAAP directly interact with the lysosomal enzymes CTSB and CTSL. Using Co-IP, we further confirmed that IAAP interacts with the CTSB and CTSL precursors (Figure 3A). IAAP inhibited CTSB and CTSL maturation in a time-dependent manner (Figure 3B). Interestingly, the knockdown of *CTSB* increased the expression of CTSL in a feedback manner, and the protein levels of LC3B remained unchanged, whereas the level of SQSTM1 decreased (Figure 3C). However, the knockdown of CTSL resulted in a significant accumulation of the autophagy-related proteins LC3B and SQSTM1 (Figure 3C). These results correspond to a study conducted by Xu et al.,^40^ suggesting that IAAP primarily affects lysosomal activity by inhibiting CTSL instead of CTSB.

We further examined the effect of IAAP on CTSL activity in BMDM, HBE, and THP-1 cells and validated the role of IAAP using a specific CTSL inhibitor, SID26681509.

SID26681509 significantly suppressed CTSL activity but did not affect CTSS activity, suggesting its specificity (Figures S3A–S3C). Our results showed that IAAP markedly inhibited CTSL activity, similar to that of SID26681509 (Figure 3D). We observed that treatment with SID26681509 also led to autolysosome accumulation (Figure S3D) and attenuated the degradation of EGFR as the concentration gradient increased (Figure S3E), consistent with the effects of IAAP. Next, we confirmed by Co-IP that IAAP interacted with the precursor of CTSL stimulated with LPS (Figure S3F). These results indicate that IAAP specifically binds to CTSL and suppresses its activity.

Discovery Studio was used to calculate the affinity between CTSL and IAAP based on an efficient optimization algorithm for the scoring function. As shown in Figures 3E and S3G, the ligand could pass through the entrance of the enzyme and substrate cavity to replace the water molecules in the active sites and fit the narrow and large CTSL-binding pocket well, thereby generating inhibitory activity. The dimethylamino and azido groups located in the entrance cavity interacted with GLU 63 establishing an attractive force of charge. The 1-methyl-indole fragment occupied the substrate cavity and formed pi-alkyl interactions with the lipophilic residues ALA 135, ALA 214, LEU 698, and MET 161. Moreover, CYS 25 and MET 70 established two Pi-sulfur interactions with the pyrimidine ring. In addition, the two amino groups on the pyrimidine formed a hydrogen bond interaction with ASP162. The above binding mode showed important interactions between IAAP and the surrounding residues. IAAP can bind to the active site of CTSL and contains strategically placed electrophilic warheads that trap the nucleophilic CYS 25 residue for activity. Collectively, these data indicate that IAAP targets and restrains lysosomal CTSL maturation, thereby inhibiting lysosomal degradation activity.

### Cathepsin L is elevated in patients with acute respiratory distress syndrome and in the murine model of lipopolysaccharide-induced acute lung injury

**Figure 4:**
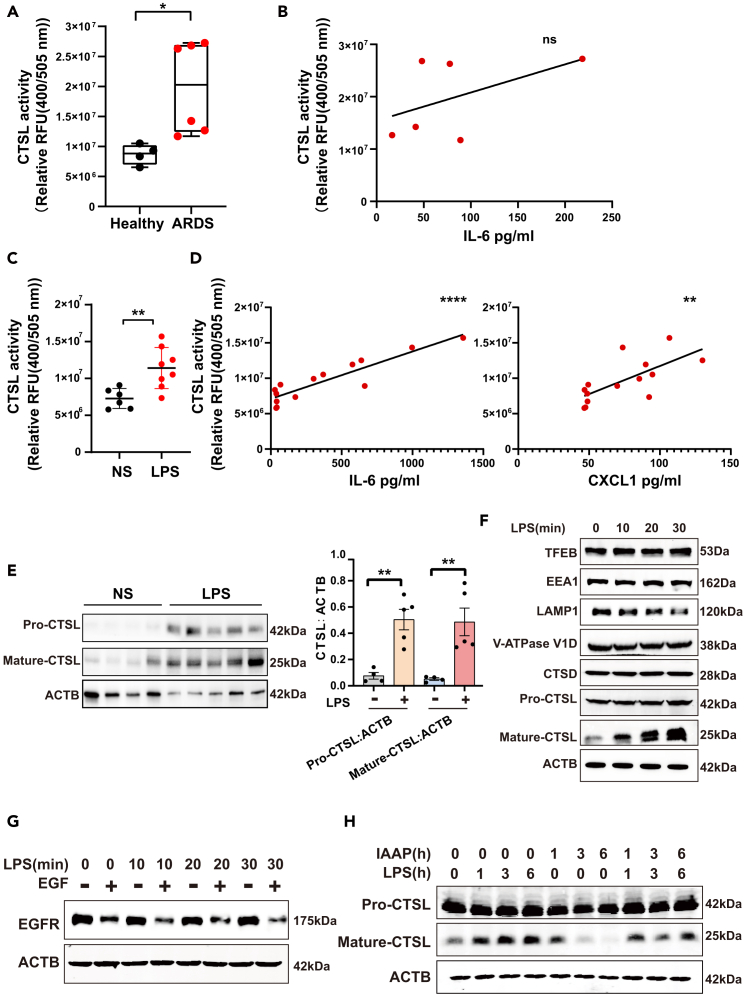
CTSL is elevated in patients with ARDS and in the murine model of LPS-induced ALI (A) CTSL activity in the BALF from healthy controls (*n* = 4) and patients with ARDS (*n* = 6). (B) Spearman’s rank correlation analysis of the relationship between CTSL activity and the levels of IL-6 in the BALF from patients with ARDS (*n* = 6). (C) CTSL activity in the BALF from mice, n (NS) = 6, n (LPS) = 8. (D) Spearman’s rank correlation analysis of the relationship between CTSL activity and the levels of IL-6 or CXCL1 in the BALF from mice, n (NS) = 6, n (LPS) = 8. (E) CTSL protein levels in the lung tissue were examined using Western blotting, n (NS) = 4, n (LPS) = 5. (F) HBE cells were treated with 100 μg/mL LPS at different time points, and the levels of TFEB, EEA1, LAMP1, V-ATPase V1D, CTSD, and CTSL were examined using Western blotting. (G) Western blot analysis of EGFR after EGF incubation in cells with 100 μg/mL LPS treatment at different time points. (H) HBE cells were treated with LPS (100 μg/mL) or IAAP (10 μM) at different time points, and the CTSL protein level was examined using Western blotting. All the quantitative data are presented as mean ± SEM. Differences between the two groups were identified using the Student’s t test. Correlations were analyzed using Spearman’s correlation analysis. ∗*p* < 0.05, ∗∗*p* < 0.01, and ∗∗∗∗*p* < 0.0001; ns, *p* > 0.05.

To explore whether CTSL is associated with the modulation of ARDS, we first assessed the status of CTSL in ARDS by assessing CTSL activity in the BALF samples from patients with ARDS and healthy controls. CTSL activity significantly increased in the BALF of patients with ARDS (Figure 4A). However, there was only a trend in Spearman’s rank correlation analysis of the relationship between CTSL activity and the levels of IL-6 but not statistically significant (Figure 4B). In the murine model of LPS-induced ALI, CTSL activity was also enhanced in the BALF (Figure 4C). Notably, CTSL activity was significantly correlated with the levels of the inflammatory cytokines IL-6 and CXCL1 in the BALF (Figure 4D). We also examined CTSS activity. The results showed that CTSS activity was increased in the BALF of mice with LPS-induced ALI which is consistent with previous research,^41^ but no increase was observed in the human BALF (Figure S4). Western blot analysis showed that the CTSL protein level was significantly increased in the lung tissue (Figure 4E). We further observed that LPS stimulation significantly increased the mature protein levels of CTSL without affecting lysosomal biosynthesis and acidification *in vitro*, whereas the protein level of LAMP1 declined (Figure 4F). LPS stimulation enhanced lysosomal degradation activity (Figure 4G). Western blot analysis demonstrated that the LPS-induced mature protein levels of CTSL were time-dependently suppressed by IAAP (Figure 4H). These results suggested that an increase in mature CTSL may contribute to the development of ARDS.

### Pharmacological inhibition of cathepsin L attenuates lipopolysaccharide-induced inflammatory response and acute lung injury

**Figure 5:**
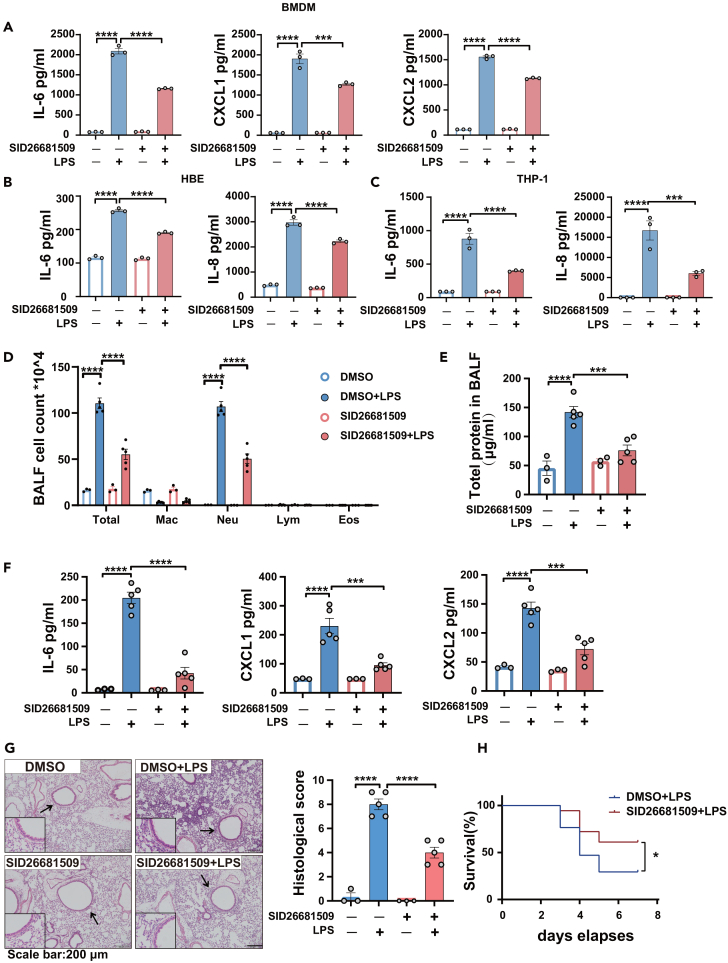
Pharmacological inhibition of CTSL attenuates LPS-induced inflammatory response and ALI (A) BMDM were stimulated with LPS (100 ng/mL), or treated with SID26681509 (5 μM) for 6 h. Cells were then harvested to analyze secreted IL-6, CXCL1, and CXCL2 proteins in cell culture supernatants using ELISA, *n* = 3 each group. (B) HBE were stimulated with LPS (100 μg/mL) or treated with SID26681509 (1 μM) for 24 h. Cells were then harvested for analyzing secreted IL-6 and IL-8 proteins in cell culture supernatants using ELISA, *n* = 3 each group. (C) THP-1 were stimulated with LPS (100 ng/mL), or treated with SID26681509 (5 μM) for 6 h, Cells were then harvested for analyzing secreted IL-6, IL-8 proteins in cell culture supernatants using ELISA, *n* = 3 each group. (D) Mice were treated by intraperitoneal injection with SID26681509 (20 mg/kg) 1h before pentobarbital injection and then with LPS (in 50 μL saline) at a dose of 5 mg/kg or NS though intratracheal administration. Mice were sacrificed 24 h after LPS administration for analysis. Total and differential cell counts in the BALF from mice, n (DMSO) = 3, n (LPS) = 5, n (SID26681509) = 3, and n (SID26681509 + LPS) = 5. (E) Total protein concentration in the BALF. (F) Expression of IL6, CXCL1, and CXCL2 in the BALF. (G) Representative images of lung tissue with HE staining 24 h post-LPS challenge and histological inflammatory scores. Scale bar: 200μm. (H) For the survival assay, mice were treated intratracheally administered LPS (20 mg/kg) on day 0. Mice received an intraperitoneal injection of SID26681509 (20 mg/kg) or PBS 1 h before intratracheal instillation on day 0 and were injected with the drugs twice on day 3. Survival rates of mice after the intratracheal administration of LPS with or without IAAP intraperitoneal injection for 24 h. n (DMSO + LPS) = 17, n (SID26681509 + LPS) = 13. DMSO was used as the vehicle control. All the quantitative data are presented as mean ± SEM and differences were identified using one-way ANOVA. The log rank (Mantel-Cox) test was used to analyze survival rates. ∗*p* < 0.05, ∗∗∗*p* < 0.001, and ∗∗∗∗*p* < 0.0001.

Next, we investigated whether CTSL is involved in the regulation of LPS-induced inflammatory responses using pharmacological inhibitors. The broad-spectrum cysteine protease inhibitor, E64D (Figures S5A and S5C), and the CTSL-selective inhibitor, SID26681509, significantly reduced LPS-induced inflammation (Figures 5A–5C), whereas the CTSB-selective inhibitor, CA074-ME, had little effect *in vitro* (Figures S5B and S5D). In addition, both IAAP and SID26681509 attenuated LPS-induced inflammatory response in BMDM from *LC3B*^−/-^ mice and *LysM*^*Cre*^*-ATG5*^*flox/flox*^ mice (Figures S5E and S5F). We investigated the therapeutic potential of CTSL-selective inhibitors in a murine model of LPS-induced ALI. SID26681509 treatment significantly alleviated LPS-induced lung inflammation (Figures 5D–5F). Histopathological examination of lung sections revealed that SID26681509 significantly decreased inflammatory cellular infiltration (Figure 5G), whereas SID26681509-treated mice survived longer than the control group after LPS administration (Figure 5H). These results suggest that CTSL is a key regulator of the LPS-induced inflammatory response, and IAAP suppresses the LPS-induced inflammatory response through CTSL instead of autophagy.

### Conditional deletion of cathepsin L in myeloid cells significantly ameliorated lipopolysaccharide-induced acute lung injury in mice

**Figure 6:**
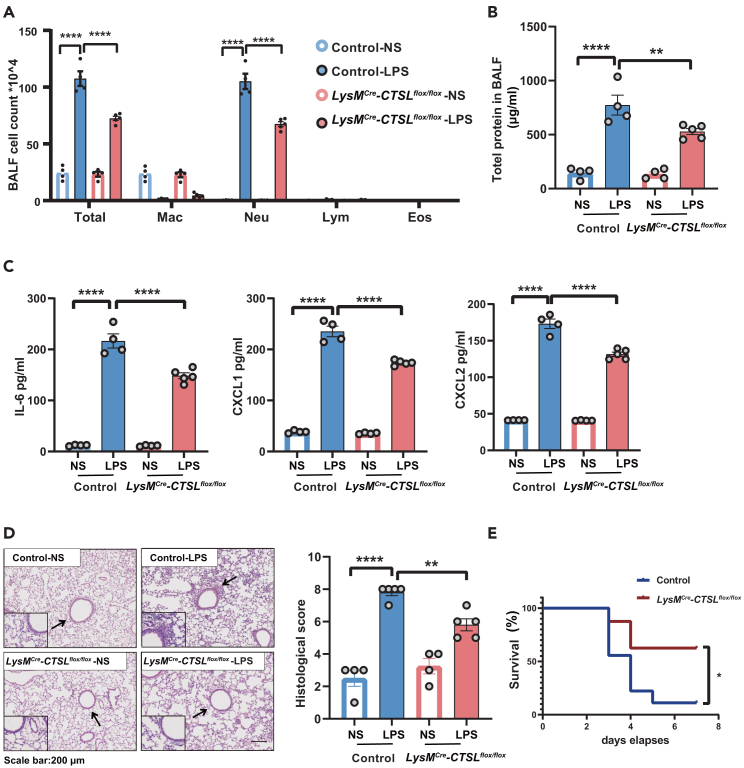
Conditional deletion of CTSL in myeloid cells significantly ameliorated LPS-induced ALI in mice (A) Mice were intratracheally treated with the LPS (in 50 μL saline) at a dose of 5 mg/kg or NS. Mice were then sacrificed 24 h after LPS administration for analysis. Total cell counts and differential cell counts in the BALF from mice, n (control-NS) = 4, n (control-LPS) = 4, n (*LysM*^*Cre*^-*CTSL*^*flox/flox*^-NS) = 4, n (*LysM*^*Cre*^-*CTSL*^*flox/flox*^ -LPS) = 5. (B) Total protein concentration in BALF. (C) Expression of IL6, CXCL1, and CXCL2 in the BALF. (D) Representative images of lung tissue with HE staining at 24 h post LPS challenge and histological inflammatory scores. Scale bar: 200 μm. (E) For surviving assay, mice were treated intratracheally with LPS (20 mg/kg) on day 0. Survival rates of mice after the intratracheal administration of LPS with or without IAAP intraperitoneally for 24 h. n (control -LPS) = 9, n (*LysM*^*Cre*^-*CTSL*^*flox/flox*^-LPS) = 8. All the quantitative data are presented as mean ± SEM and differences were identified using one-way ANOVA. The log rank (Mantel-Cox) test was used to analyzed survival rates. ∗*p* < 0.05, ∗∗*p* < 0.01, ∗∗∗∗*p* < 0.0001; ns, *p* > 0.05.

To further explore the role of CTSL in ALI, myeloid cell-specific CTSL deletion mice (*LysM*^*Cre*^-*CTSL*^*flox/flox*^ mice) were used. The deletion of CTSL in myeloid cells was achieved by crossing floxed CTSL mice (*CTSL*^*flox/flox*^) with *LysM*^*Cre*^ mice (Figure S6). In the LPS-induced ALI mouse model, conditional deletion of the myeloid cell CTSL significantly decreased the total cell count, neutrophil count, and protein concentration in the BALF (Figures 6A and 6B). In addition, the levels of inflammatory factors in the BALF, including IL-6, CXCL1, and CXCL2, were markedly reduced in the BALF from *LysM*^*Cre*^-*CTSL*^*flox/flox*^ mice compared to those in control mice (Figure 6C). Consistently, mild inflammatory cell infiltration was observed in the lung tissues of *LysM*^*Cre*^-*CTSL*^*flox/flox*^ mice (Figure 6D), and the survival rate of *LysM*^*Cre*^-*CTSL*^*flox/flox*^ mice was significantly increased after LPS exposure compared to that of control mice (Figure 6E). These results suggested that CTSL facilitated LPS-induced inflammatory responses.

### Cathepsin L is essential for lipopolysaccharide-induced K63-linked polyUb binding of nuclear factor kappa B essential modulator by degrading A20

**Figure 7:**
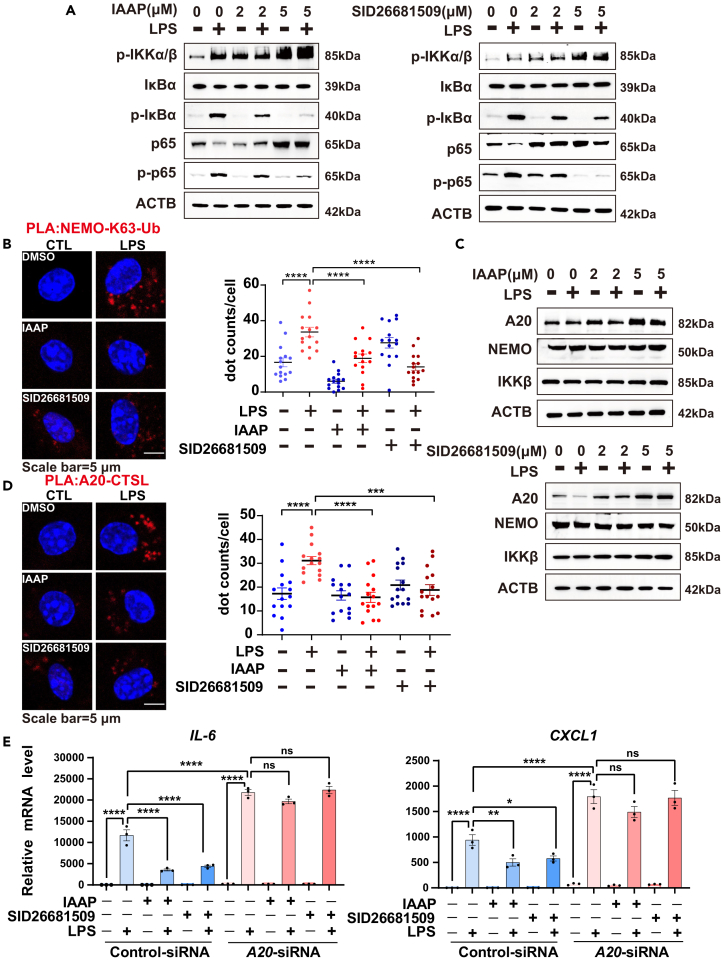
CTSL regulates the LPS-induced K63-linked polyUb binding of NEMO by degrading A20 (A) BMDM cells were treated with IAAP or SID26681509 for 6 h, then stimulated with LPS (100 ng/mL) for 5 min, the levels of *p*-IKKα/β, IκBα, *p*-IκBα, p65, p-p65, and ACTB were examined using Western blotting. (B) BMDM cells were treated with IAAP or SID26681509 for 6 h, then stimulated with LPS (100 ng/mL) for 5 min. BMDM was analyzed for the spatial approximation of IKKγ with K63-linked polyUb components using PLA. Red, proximity ligation-positive signals. Scale bars: 5 μm. The quantification shown on the right represents the fluorescence, *n* = 15. (C) BMDM cells were treated with IAAP or SID26681509 for 6 h, then stimulated with LPS (100 ng/mL) for 5 min, the levels of A20, NEMO, IKKβ, and ACTB were examined using Western blotting. (D) BMDM cells were analyzed for the spatial approximation of A20 with CTSL components using PLA. Red, proximity ligation-positive signals. Scale bars: 5 μm. Quantification shown on the right represents the fluorescence, *n* = 15. (E) BMDM cells were transfected with control-, *A20*-siRNA for 24 h. Then cells were treated with IAAP (5 μM) or SID26681509 (5 μM) for 6 h, and then stimulated with LPS (100 ng/mL) for 5 min. Cells were then harvested for analyzing the mRNA expression of IL-6 and CXCL1 using qRT-PCR, *n* = 3 in each group. DMSO was used as the vehicle control. All the quantitative data are presented as mean ± SEM and differences were identified using one-way ANOVA. ∗*p* < 0.05, ∗∗*p* < 0.01, ∗∗∗*p* < 0.001, and ∗∗∗∗*p* < 0.0001; ns, *p* > 0.05.

After clarifying the protective effect of the CTSL inhibitors on the LPS-induced inflammatory response, we further explored the regulatory mechanism of CTSL. Given that the NF-κB signal pathway was the key mechanism to mediate LPS-induced inflammation, we examined whether CTSL-selective inhibitors affect the NF-κB signal pathway. CTSL inhibitors concentration-dependently reduced the phosphorylation of IκBα and p65, whereas it was quite unexpected that CTSL inhibitors led to a significant accumulation of the phosphorylated IKKα/β in BMDM and HBE cells (Figures 7A, S7A, and S7B). The IκB kinase complex (IKK) consists of IKKα, IKKβ, and NEMO. In response to stimulation by LPS, K63-linked polyUb recruits the IKK complex by binding to NEMO, and then IKK is activated to phosphorylate IκBα, leading to NF-κB activation.^18^^,^^19^^,^^42^ The proximity ligation assay (PLA) showed strong signals for interactions between NEMO and K63-linked polyUb upon LPS administration, whereas CTSL inhibitors reduced their associations (Figure 7B), suggesting that the ubiquitination of NEMO was modulated by CTSL in the context of LPS-induced inflammatory responses.

A20 is a deubiquitinating enzyme that removes K63-linked polyUb from the target protein and thereby negatively regulates NF-κB.^42^^,^^43^ Consistent with previous reports, the PLA indicated that LPS increased the colocalization of A20 and LAMP1, suggesting that lysosomes are the primary A20 degradation pathway (Figure 7D).^44^^,^^45^ We found that LPS treatment led to a decrease in A20 levels, which was significantly reversed by the CTSL inhibitors (Figures 7C, S7A, and S7B). Similar results were observed that genetic knockout of CTSL effectively reduced the protein levels of *p*-IκBα and p-p65, but rescued A20 degradation in response to LPS, and elevated *p*-IKKα/β protein (Figures S7C). Thus, we hypothesized that CTSL directly degrades A20 in lysosomes. As expected, we observed that the association between CTSL and A20 increased after LPS treatment, and was significantly reduced by CTSL inhibitors (Figure 7D). We also showed that LPS stimulation promoted the binding of A20 to CTSL (Figure S7D), and CTSL inhibitors barely suppressed LPS-induced inflammation when *A20* was knocked down, suggesting that CTSL-mediated LPS-induced inflammatory responses are dependent on A20 (Figure 7E). These experiments collectively indicated that the LPS-induced upregulation of CTSL targeted to degrade A20 in lysosomes and regulated the K63-linked polyUb binding of NEMO, thereby activating the NF-κB pathway.

## Discussion

In this study, we describe IAAP as a potent small-molecule inhibitor that targets the degradation activity of the lysosomal enzyme CTSL. We determined that CTSL is involved in the regulation of LPS-induced NF-κB activation and pulmonary inflammation. Mechanistically, LPS triggers the maturation of CTSL and enhances its lysosomal activity, leading to the degradation of A20, and results in a significant accumulation of K63-polyubiquitinated NEMO, promoting the kinase activity of the IKK complex and subsequently NF-κB activation. Pharmacological inhibition and genetic knockout of CTSL rescued LPS-induced degradation of A20 reduced K63-polyubiquitinated NEMO and eventually attenuated LPS-induced inflammatory responses.

ALI/ARDS is an acute inflammatory disease associated with high morbidity and mortality rates. Curative therapy for ALI/ARDS remains a significant challenge in critical care medicine and no specific pharmacotherapy has been proven to be effective. We synthesized the small-molecule compound IAAP as a candidate anti-inflammatory drug.^38^ Our current study showed that IAAP ameliorates LPS-induced inflammatory responses *in vitro* and ALI *in vivo*. Using LC-MS/MS and KEGG pathway enrichment analysis, we showed that IAAP impaired the autophagy-lysosome pathway by reducing the mature protein levels of lysosomal cathepsins, CTSB, and CTSL. Knockdown of *CTSB* enhanced CTSL expression, and CTSL overexpression promoted autophagosome degradation as reported previously,^46^ suggesting that IAAP exerts its effect through CTSL instead of through CTSB. Our previous study validated that LPS inhibits autophagy by activating mTORC1, which then activates the NF-κB signaling to promote inflammation in ALI.^16^ We next determined that both IAAP and SID26681509 attenuated LPS-induced inflammatory response in BMDM from *Lc3b*-deficient and *LysM*^*Cre*^*-Atg5*^flox/flox^ mice. Thus, our results suggest that IAAP regulates the LPS-induced inflammatory response through CTSL instead of autophagy and that CTSL may play a critical role in the regulation of the LPS-induced inflammatory response.

The lysosome serves not only as the center for degradation and metabolism but also as a hub for several signaling pathways. Most lysosomal studies have focused on metabolic disorders, neurodegenerative diseases, and tumors.^21^^,^^47^^,^^48^ In recent years, lysosomal enzymes, particularly cathepsins, have received increasing attention as potential targets for the treatment of inflammatory diseases. LPS or IFN-γ increases cathepsins expression in macrophages.^49^^,^^50^ CTSB, CTSL, and CTSS are overexpressed in lung tissues of patients with cystic fibrosis.^51^^,^^52^ CTSB could enter the cytoplasm and activate NLRP3 inflammasomes, leading to the maturation and secretion of cytokines such as IL-1β, IL-6, and TNFα.^53^ In patients with ALI/ARDS, McKelvey et al. have reported that CTSS is elevated in the lungs of patients with ALI/ARDS and in animal models of ALI/ARDS. CTSS may partially mediate its pathogenic effects via protease-activated receptor-1.^41^ This study investigated the status and role of CTSL in patients with ALI and ARDS. CTSL levels were elevated in the BALF samples from patients with ARDS and in a murine model of ALI. LPS increased the mature protein levels of CTSL without affecting lysosomal biosynthesis, acidification, or maturation. It is worth emphasizing that the maturation of CTSL was associated with LPS-induced inflammation. Using the pharmacological inhibition of cathepsins and myeloid cell-specific CTSL deletion in mice (*LysM*^*Cre*^-*CTSL*^*flox/flox*^ mice), we confirmed that CTSL plays a critical role in the regulation of LPS-induced inflammation.

In addition, we examined the effects of *CTSL*-siRNA on LPS-induced inflammation *in vitro* and determined that the knockdown of *CTSL* barely affected the LPS-induced inflammatory response (data not shown). One plausible explanation is that CTSL is a dual-function molecule, whose functional switch is regulated by its maturation state similar to CTSD.^54^ Pro-CTSL may have important consequences for normal cellular physiological processes, whereas *CTSL-*siRNA reduced both pro-CTSL and mature-CTSL protein levels instead of inhibiting their enzymatic activity as CTSL inhibitors do.

The canonical NF-κB pathway responds to diverse immune stimuli such as LPS and TNFα, resulting in sharp but transient NF-κB activation.^55^ Yang et al. have reported that the increased CTSB decreased TNFα-mediated NF-κB activation in HDAC3-deficient macrophages as a scissor for the significant lysosomal degradation of RIP1.^56^ Similarly, we showed that CTSL plays a critical role in regulating NF-κB activation and function but has a mechanism distinct from that of CTSB. In the canonical NF-κB pathway, IκBα is a negative regulator downstream of the NF-κB signaling. Upon activation, IκBα is phosphorylated by the IKK complex, after which IκBα is degraded, leading to the translocation of NF-κB into the nucleus. There, it activates the expression of numerous genes, including those involved in immune and inflammatory responses. The IKK complex consists of IKKα, IKKβ, and NEMO. Phosphorylated IKKβ and K63-polyubiquitinated NEMO are required for the kinase function.^18^^,^^19^ A20 is an important ubiquitin editing enzyme involved in deubiquitination modifications such as K63-linked polyUb in NF-κB signaling.^57^^,^^58^ Following LPS stimulation, A20 is bound by lysosomal-associated protein transmembrane 5 (LAPTM5) and transported to the lysosome for degradation, thereby activating the NF-κB signaling.^44^ Notably, our study determined that the LPS-induced up-regulation of CTSL is targeted to degrade A20 in the lysosomes and decrease K63-linked polyUb binding of NEMO to maintain the IKK complex activation. In addition, we showed here that the recruitment of the IKK complex and phosphorylation of IKKβ could happen independent of the K63-linked polyUb binding of NEMO. CTSL-selective inhibitor effectively leads to the accumulation of phosphorylated IKKβ, however, a reduction of K63-linked polyUb binding of NEMO, thereby inhibiting the activation of NF-κB signaling and the LPS-induced inflammation and ALI.

In summary, the present study showed that IAAP targets lysosomal CTSL activity to suppress the LPS-induced ALI through deubiquitinase A20 to modulate the NF-κB signaling. Our findings suggest that CTSL is a valuable therapeutic target and IAAP can serve as a potential lead compound for the development of new drugs for ALI.

### Limitations of the study

This study has several limitations. First, although we have utilized the most reliable research methods available to assess CTSL activity, there is some controversy regarding the substrate used for detecting cathepsin activity. This is a problem within the field that requires further in-depth study. Second, the precursor and mature forms of CTSL may exert differential effects on cellular functions, including the inflammatory response, which warrant further investigation.

## Resource availability

### Lead contact

Further information and requests for resources and reagents should be directed to and will be fulfilled by the lead contact, Zhihua Chen (Email: zhihuachen@zju.edu.cn).

### Material availability

This study did not generate new, unique reagents.

### Data and code availability

• The supplemental information contains most results. Additional results, including data going into all figures and tables, are available from the lead contact.
• This article does not report the original code.
• Any additional information needed to reanalyze the data reported in this study may be requested from the lead contact.

## Acknowledgments

This work was supported by the Major Project from the 10.13039/501100004731Natural Science Foundation of Zhejiang Province (LD21H010001 to ZC), and the General Projects (31970826 to ZC, 82270086 to GZ, 22277108 to XZ, 21877099 to XZ) from the 10.13039/501100001809National Natural Science Foundation of China.

We thank the technical support of the Core Facilities, Zhejiang University School of Medicine. We thank Chenyu Yang and Beibei Wang in the Centre of CryoElectron Microscopy (CCEM), 10.13039/501100004835Zhejiang University for their technical assistance on Transmission Electron Microscopy. We thank the staff members of the Mass Spectrometry System at the National Facility for Protein Science in Shanghai (NFPS), Zhang jiang Lab, SARI, China for providing technical support and assistance in data collection and analysis.

We would like to dedicate this article to Huahao Shen, who unfortunately passed away just before the article was submitted for publication. Shen played an essential role in the research described here and he is greatly missed.

## Author contributions

Z.C., H.S., G.Z., and X.Z. designed and supervised the study; S.Y., K.C., J.Y., Z.J., M.Z., Z.L., Y.W., Y.Z., and X.F. performed experiments; T. C. and W. W. performed the synthesis of the compound IAAP and Z. M. performed the docking analysis of CTSL and IAAP. Y.Y., N.X., B.T., and N.L. collected human samples. S.Y., K.C., M.Z., and Z.M. prepared figs; S.Y., Z.M., and Z.C. drafted the article; S.Y., W.L., F.Y., H.S., X.Z., G.Z., and Z.C. analyzed data and revised article. All authors approved the final article.

## Declaration of interests

The authors declare no competing interests.

## STAR★Methods

### Key resources table

**Table:** 

REAGENT or RESOURCE	SOURCE	IDENTIFIER
**Antibodies**		

ACTB	ABclonal	Cat#AC026; RRID: AB_2768234
A20	Cell Signaling Technology	Cat#5630; RRID: AB_10698880
CTSD	BD Biosciences	Cat#610800; RRID: AB_398119
CTSB	Cell Signaling Technology	Cat#31718; RRID: AB_2687580
CTSL	Sigma-Aldrich	Cat#C4618; RRID: AB_1078407
EGFR	Abcam	Cat#ab52894; RRID: AB_869579
EEA1	Santa Cruz	Cat#sc-137130; RRID: AB_2246349
TFEB	Abcam	Cat#ab122910; RRID: AB_10901928
IKKα/β	Abcam	Cat#EPR16628; RRID: AB_2924430
IKKγ/NEMO	Cell Signaling Technology	Cat#2695; RRID: AB_2124826
IκBα	Cell Signaling Technology	Cat#4814; RRID: AB_390781
LAMP1	Santa Cruz	Cat#sc-20011; RRID: AB_626853
LC3B	Sigma-Aldrich	Cat#L7543; RRID: AB_796155
*p*-IKK α/β	Abcam	Cat#ab194528
p65	Cell Signaling Technology	Cat#6956; RRID: AB_10828935
p-p65	Cell Signaling Technology	Cat#3033; RRID: AB_331284
P-IκBα	Cell Signaling Technology	Cat#2859; RRID: AB_561111
SQSTM1	Cell Signaling Technology	Cat#88588; RRID: AB_2800125
K63-linkage Specific Polyubiquitin (D7A11)	Cell Signaling Technology	Cat#5621; RRID: AB_10827985
V-ATPase D	Santa Cruz	Cat#sc-166218; RRID: AB_2062530
Goat anti-Mouse IgG (H + L) Secondary Antibody	Thermo Fisher Scientific	Cat#31430; RRID: AB_228307
Goat anti-Rabbit IgG (H + L) Secondary Antibody	Thermo Fisher Scientific	Cat#31460; RRID: AB_228341
Goat anti-Rabbit IgG (H + L) Highly Cross-Adsorbed Secondary Antibody, Alexa Fluor™ 488	Thermo Fisher Scientific	Cat#A-11034; RRID: AB_2576217
Goat anti-Mouse IgG (H + L) Cross-Adsorbed Secondary Antibody, Alexa Fluor™ 488	Thermo Fisher Scientific	Cat#A-11001; RRID: AB_2534069
Goat anti-Rabbit IgG (H + L) Cross-Adsorbed Secondary Antibody, Alexa Fluor™ 555	Thermo Fisher Scientific	Cat#A-21428; RRID: AB_2535849
Rabbit anti-Mouse IgG (H + L) Cross-Adsorbed Secondary Antibody, Alexa Fluor™ 555	Thermo Fisher Scientific	Cat#A-21427; RRID: AB_2535848

**Chemicals, peptides, and recombinant proteins**		

Lipopolysaccharide	Sigma-Aldrich	Cat#L2880
EGF	Thermo Fisher Scientific	Cat#E3480
CA074-ME	MedChemExpress	Cat#HY-100350
SID26681509	MedChemExpress	Cat#HY-103353
Cycloheximide	MedChemExpress	Cat#HY-12320
Bafilomycin A1	MedChemExpress	Cat#HY-100558
LysoTracker™ Red DND-99	Thermo Fisher Scientific	Cat# L7528
Dynabead™ MyOne™ Streptomycin C1	Thermo Fisher Scientific	Cat#65001
BeyoMag™ Protein A + G Magnetic Beads	Beyotime Biotechnology	Cat#P2108

**Critical commercial assays**		

GenMute™ siRNA Transfection Reagent	SignaGen Laboratories	Cat#SL100568
DNA Transfection reagent	SignaGen Laboratories	Cat#SL100688
BCA Assay	Thermo Fisher Scientific	Cat# 23225
DAPI-containing Fluoromount-G™	SouthernBiotech	Cat#0100-20
Cathepsin L Activity Fluorometric Assay Kit	Abcam	Cat#ab65306
Duolink *in Situ* Red Starter Kit Mouse/Rabbit	Sigma-Aldrich	Cat#DUO92101
Human IL-6 ELISA	Dakewe	Cat#1110602
Human IL-8 ELISA	Dakewe	Cat#1110802
Mouse CXCL1 ELISA	MultiSciences, LiankeBio	Cat#70-EK296/2-96
Mouse CXCL2 ELISA	MultiSciences, LiankeBio	Cat#70-EK2142/2-96
Mouse IL-6 ELISA	MultiSciences, LiankeBio	Cat#70-EK206/3-96

**Experimental models: Cell lines**		

HBE	ATCC	Cat#CRL-2741; RRID:CVCL_3695
HEK293T	ATCC	Cat#CRL-11268 (RRID:CVCL_1926)
THP-1	ATCC	Cat#TIB-202 (RRID:CVCL_0006)

**Oligonucleotides**		

Control	Santa cruz	Cat#sc-37007
*CTSB* (human)	Santa cruz	Cat#sc-36462
*CTSL* (human)	Santa cruz	Cat#sc-44327
*A20* (mouse)	GenePharma	Cat#A01001
Primers for recombinant DNA	This paper	see Table S3

**Software and algorithms**		

GraphPad Prism (version8.0)	GraphPad software	https://www.graphpad.com/
FlowJo	FlowJo	https://www.flowjo.com/
ImageJ v1.52q	Wayne Rasband	https://imagej.nih.gov/ij/index.html
FV31S-SW	Olympus	N/A
BioRad Image Lab Software-6.1	LI-COR Biosciences	https://www.bio-rad.com

### Experimental model and subject details

#### Human samples

We collected human BALF samples (10mL–15mL per person) from four healthy controls and six patients with ARDS recruited from the clinical population at the Department of Respiratory and Critical Care Medicine and the Department of Critical Care Medicine, Second Affiliated Hospital of Zhejiang University School of Medicine from 2022 to 2023. The diagnosis of ARDS was based on the guidelines for the diagnosis and treatment of ALI/ARDS (diagnosed by the Congress of Chinese Society of Critical Care Medicine, which shows no differences in the diagnosis of ARDS compared with ATS/ESICM/SCCM guidelines^59^). ARDS patients over the age of 18 were included, and sex- and age-matched healthy subjects were enrolled as controls. No differences in age or sex were found between the healthy control group and ARDS group (Table S1). This study was approved by the ethics committees of The Second Affiliated Hospital of Zhejiang University, School of Medicine (approval No. IR2022686 to Huahao Shen). All patients wrote the informed consent. Additional clinical information can be found in Tables S1 and S2.

#### Mice

*CTSL*^*flox/flox*^ mice (C57BL/6 background) were purchased from Cyagen Biosciences (Jiangsu, China). *LysM*^*Cre*^ mice (C57BL/6 background) were provided by Dr. G. Feng (University of California at San Diego, CA). *LC3B*^*−/-*^ mice and *ATG5*^*flox/flox*^ were obtained from Jackson Laboratory. *LysM*^*Cre*^-*CTSL*^*flox/flox*^ mice were generated by crossing the *CTSL*^*flox/flox*^ mice with *LysM*^*Cre*^ mice. *LysM*^*Cre*^-*CTSL*^*flox/flox*^ mice and littermate control mice (*CTSL*^*flox/flox*^) were used for the experiments. *LysM*^*Cre*^- *ATG5*^*flox/flox*^ mice were generated by crossing the *ATG5*^*flox/flox*^ mice with *LysM*^*Cre*^ mice.

C57BL/6 mice (male, wild-type, 6 to 8 weeks old, 18–20 g) were purchased from the Animal Center of Slaccas (Shanghai, China). Age- and sex-matched mice were randomized to different groups.

All mice were maintained in a specific pathogen-free facility. All experimental protocols were approved by the Ethical Committee for Animal Studies at Zhejiang University (ZJU20210302).

#### ALI mouse models

To establish a mouse model of ALI, mice were treated intraperitoneally with 1% pentobarbital in PBS (90 mg/kg), and then with LPS (in 50 μL saline) at a dose of 5 mg/kg or normal saline (NS) through intratracheal administration for 24 h. IAAP (20 mg/kg) or SID26681509 (20 mg/kg) were dissolved in dimethyl sulfoxide (DMSO). Mice were treated by intraperitoneal injection with IAAP (in 100 μL DMSO), or SID26681509 (in 100 μL DMSO) 1h before LPS administration. DMSO serves as the vehicle control. Mice were sacrificed 24 h after LPS administration for analysis.

#### Cell lines

Human bronchial epithelial (HBE) cells were purchased from American Type Culture Collection (ATCC, Cat#CRL-2741, RRID: CVCL_3695), as well as Tohoku Hospital Pediatrics-1 (THP-1) cells (ATCC, Cat#TIB-202, RRID: CVCL_0006). HBE and THP-1 were cultured in RPMI 1640 (Sigma-Aldrich, Cat#R8758) supplemented with 10% fetal bovine serum (FBS, Gibco, Cat#10082147) and 1% penicillin-streptomycin (Beyotime Biotechnology, Cat#C0222) in 5% CO2 at 37°C. Cells were subjected to further analysis.

#### Isolation and culture of BMDM

Bone marrow-derived macrophages (BMDM) were obtained from the bone marrow of 4–6 weeks-old mice. Age- and sex-matched mice were sacrificed via cervical dislocation and soaked in 75% ethanol. The femurs and tibias were harvested and the bone marrow cells from all bones were flushed out. After being filtered through a 40 μm cell strainer and centrifuging for 5 min at 400 g, erythrocytes were eliminated using RBC Lysing Buffer (Sigma-Aldrich). Bone marrow cells were plated on 10 cm dishes in RPMI1640 (Sigma-Aldrich, Cat#R8758) supplemented with 20 ng/mL Macrophage colony stimulating factor 1(novoprotein, Cat#CB34), 10% FBS, and 1% penicillin-streptomycin in 5% CO2 at 37°C. The medium was replaced on day 3 and further experiments were performed with proliferative nonactivated BMDMs on day 5. Cells were then subjected to further analysis.

### Method details

#### Synthesis of IAAP

2-Azido-N-(2-((2-(dimethylamino) ethyl) (methyl)amino)-4-methoxy-5-((4-(1-methyl-1H-indol-3-yl) pyrimidin-2-yl) amino) phenyl) acetamide was synthesis as described in Chen et al.^38^ Briefly, 2,4-dichloropyrimidine was treated with 1-methylindole in the presence of AlCl3, and then the intermediate 1 was synthesized via aromatic amination on the 2-position of pyrimidine. Intermediate 2 was generated through nucleophilic substitution of intermediate 1 with various aliphatic secondary amines, reduction of the nitro group followed by treatment with HCl gas in EtOH. The intermediate 2 was reacted with various acyl chlorides and underwent nucleophilic substitution with sodium azide to produce IAAP.

#### MTT assay

MTT assay was performed according to the manufacturer’s instructions (Beyotime Biotechnology, Cat#C0009S). BMDM, HBE, and THP-1 cells were cultured in 96-well plates, with the edge wells were filled with 200 μL sterile PBS. Cells were treated with drugs at different concentrations as required by the experiment. Afterward, 10 μL of MTT solutions (5 mg/mL) was added to each well, and the cells were incubated for 4 h at 37°C. Subsequently, 100 μL of Formazan solvent was added to each well, followed by an additional 3-h incubation. The 96- well plate was placed in an enzyme calibrator to read the OD value of each well at 570 nm. The cell survival rate ≥70% indicates that the drug has no cytotoxicity.

#### BALF collection and analysis

Mice were sacrificed 24 h after the exposure to LPS and lavaged with 0.4 mL PBS (Solarbio, Cat#NO. P1010) by injecting into the lungs and drawing to collect cells 3 times. The BALF was used for cell counts and cell differential analysis, then the remaining BALF was centrifuged (3000 rpm for 10 min at 4°C) and the supernatant fractions were collected and stored at −80°C. The cell pellet was mildly resuspended in 200 μL PBS, and 30 μL of the suspension was spun onto glass microscope slides using the Shandon Cytospin 3 (Thermo Fisher Scientific, Waltham, MA, USA). Wright-Giemsa stain (Baso, Cat#BA-4017) was used to stain cells on glass slides, and differential counts were determined by counting 200 total cells. The supernatants were utilized for protein quantification and cytokine analyses.

Human BALF (10mL–15mL) obtained from all the enrolled patients was centrifuged (2000 rpm for 10 min at 4°C). The supernatants were kept at −80°C and utilized for evaluating CTSL activity.

#### Histological analysis

After exposure to LPS, the lungs were removed and fixed in 4% paraformaldehyde for 24 h. The lungs were embedded in paraffin for hematoxylin & eosin (H&E) analysis after fixation. The features of lung interstitial edema, hemorrhage, and neutrophil infiltration were used to determine the inflammatory score. Scores of 0 (no injury), 1 (limited injury), 2 (visible injury), and 3 (injury) could be assigned to each feature (severe injury) according to published guidelines.^60^ The combined scores from the three factors range from 0 to 9.

#### Survival rate

For surviving assay, mice were treated intratracheally with LPS (in 50 μL saline) at a dose of 20 mg/kg. IAAP (20 mg/kg) or SID26681509 (20 mg/kg) were dissolved in dimethyl sulfoxide (DMSO). Mice received an intraperitoneal injection of IAAP or SID26681509 1 h before LPS administration on day 0, and injected with drugs twice on day 3. DMSO serves as the vehicle control. The survival percentages and body weights in each group were monitored every 24 h after LPS instillation for more than 7 days. Death was seen as being equal to body weight loss of 20% or less of the starting weight.

#### Molecular docking

The predicted binding mode of IAAP in CTSL was generated by molecular docking. The X-ray structures of complexes of CTSL/Ligands were acquired from the Protein DataBank (PDB) with the accession codes 3HWN.^61^ The CDOCKER program in the Discovery Studio 4.5 software (version 4.5, BIOVIA, USA) was used to perform docking simulations. Crystallized water molecules and ligands in the active site were deleted. Chain B, chain C, and chain D in the 3HWN were deleted, and all computations were performed in chain A. All the parameters for the protein preparation, ligands minimization, ligand preparation, and docking run were set to their default values.

#### Transfection

A set of siRNAs were purchased from Santa Cruz and GenePharma. 5 × 10^4^ cells were seeded in each well of 6-well plates and grown overnight. The siRNA transfection was performed with GenMute siRNA Transfection Reagent (SignaGen Laboratories, Cat#SL100568) following the manufacturer’s protocol. According to the manufacturer’s protocol, the GFP-LC3, GFP-RFP-LC3 plasmid was transfected into cells by using PloyJet *in vitro* DNA Transfection reagent (SignaGen Laboratories, Cat#SL100688).

#### Immunoprecipitation studies

For co-immunoprecipitation experiments, HBE cells after experimental manipulations were collected and suspended in lysis buffer (Beyotime Biotechnology, Cat#P0013J) containing protease inhibitors (Roche Diagnostics GmbH, Cat#04-693-116-001) and phosphatase inhibitors (Roche Diagnostics GmbH, Cat#04-906-837-001) on ice for 30 min, followed by centrifugation at 12,000 rpm for 10 min at 4°C. Supernatant was mixed with lysis buffer plus the loading buffer as the input sample. The remaining supernatants were immunoprecipitated with Dynabead MyOne Streptomycin C1 (Thermo Fisher Scientific, Cat#65001) or BeyoMag Protein A + G Magnetic Beads (Beyotime Biotechnology, Cat#P2108) conjugated with indicated antibodies at 4°C and rotated for 3–5 h. The beads were washed with lysis buffer three times at 4°C. Then the beads were boiled with 60 μL 1 × loading buffer at 100°C for 10 min and analyzed by western blot.

#### Liquid chromatography-tandem mass spectrometry (LC-MS/MS) and KEGG enrichment analysis

The on-beads digestion samples after co-immunoprecipitation experiments were resuspended in lysis buffer and sent to the Mass Spectrometry System at the National Facility for Protein Science in Shanghai, Zhangjiang Lab, SARI, China, for protein identification. The proteins identified by LC-MS/MS were converted into unified Homo sapiens gene symbol name and Entrez ID using the Uniprot protein database (https://www.uniprot.org/). KEGG enrichment analysis was performed using the clusterProfiler package (version 3.14.3) R package. A gene was defined as differentially expressed if its fold-change value was 1 or more, with an adjusted *p* value ≤ 0.05.

#### Immunofluorescence staining

Fluorescence images were captured with the confocal microscope (Olympus, Melville, NY, USA). Cells were fixed and stained with primary antibody at 37°C for 2 h. Secondary antibody (Thermo Fisher Scientific, Cat#A-11034 and Cat#A-21427) was used to visualize the bound primary antibody. Cell micrographs were obtained using a fluorescence microscope. The relative fluorescence intensity was measured with ImageJ software, and the mean relative fluorescence intensities were normalized to the levels of control.

#### Labeling lysosomes with Lysotracker

LysoTracker red is a specific lysosomal red fluorescent probe. Lyso-Tracker Red DND-99 was purchased from Thermo Fisher Scientific. After LPS treatment at 100 mg/mL for 24 h, cells were stained with 50nM LysoTracker red (preheated at 37°C for 30min before use) at 37°C for 1 h. After staining, cells were washed with PBS twice, and cellular fluorescence was examined by confocal microscope or flow cytometry.

#### Proximity ligation assay (PLA)

Antibodies used in this assay were antecedently rigorously tested with immunofluorescence. PLA was performed according to the manufacturer’s protocol (Duolink *in Situ* Red Starter Kit Mouse/Rabbit, Sigma-Aldrich, Cat#DUO92101). Briefly, BMDM cells were seeded in a 12-well chamber slide. Cells were fixed with 4% formaldehyde for 15 min and permeabilized with 0.1% Triton X-100 in PBS for 10 min. The samples were blocked and then incubated overnight with antibodies. Subsequently, the samples were incubated with the MINUS and PLUS PLA probes corresponding to the primary antibodies used, followed by ligation with circle-forming DNA oligonucleotides and rolling-circle amplification to generate the PLA signal. Finally, the samples were mounted with DAPI-containing Fluoromount-G (SouthernBiotech). The slides were imaged with a confocal microscope. Analysis of the data was done by counting the PLA signals per cell manually.

#### EGFR degradation assay

After culture in serum-free 1640 for 5 h, cells were treated with LPS (100 μg/mL), Baf A1 (5 nM), or IAAP (5 μM) respectively for 6 h, and then incubated with 10 μM cycloheximide for another 2 h. Then, the cells were treated with 100 ng/mL EGF (Thermo Fisher Scientific, Cat#E3480) for different durations. The EGFR expression level was evaluated by cellular immunoblotting assay.

#### Semi-quantitative RT-PCR

Cells and lung homogenates were lysed with RNAiso plus (Vazyme, Cat#R401), and total RNA was extracted. By using reverse transcription reagents (Vazyme, Cat#R232), RNAs were reverse-transcribed. Then, the expression of genes was measured by quantitative real-time PCR, which was performed on a StepOne real-time PCR system (Applied Biosystems, Foster City, CA, USA) using SYBR Green Master Mix (Takara Biotechnology, Cat#DRR041A). Primers used for real-time quantitative reverse transcription-polymerase chain reaction (qRT-PCR) analysis are provided in Table S3. All protocols were performed following the manufacturer’s instructions.

#### Western blot assay

Cells and lung tissue were prepared with RIPA buffer (Beyotime Biotechnology, Cat#P0013B) containing protease inhibitors and phosphatase inhibitors. Samples were electrophoresed through 6–15% polyacrylamide gels and immunoblotted with the relevant antibodies using standard methods.

#### Enzyme-linked immunosorbent assay (ELISA)

BALF and cell culture medium samples were centrifuged at 400 g for 10 min, and the supernatant fractions were collected and stored at −80°C. The concentration of IL6 and IL8 in culture supernatants and mouse cytokines such as CXCL1, CXCL2, and IL6 in BALF supernatants, were measured by ELISA kits following the manufacturer’s protocol. ELISA kits for human IL6 (Cat#1110602), and human IL8 (Cat#1110802) were purchased from Dakewe. Mouse CXCL1 (Cat#70-EK296/2–96), mouse CXCL2 (Cat#70-EK2142/2–96), and mouse IL6 (Cat#70-EK206/3–96) were purchased from MultiSciences, LiankeBio.

#### Cathepsin L activity assay

Cathepsin L Activity Fluorometric Assay Kit (Abcam, Cat#ab65306) utilizes the preferred CTSL substrate phenylalanine-arginine (FR) labeled with fluorescent AFC (7-amino4- trifuoromethylcoumarin). The activity of lysosomal CTSL was measured using the fluorescent activity assay kit according to the manufacturer’s instructions. The fluorescence was visualized by a multifunctional microplate reader (Molecular Devices, SpectraMax iD5, CA, USA) at excitation and emission wavelengths of 400 and 505 nm.

MAGIC RED Cathepsin L Assays (Bio-rad, Cat#ict941) utilizes ICT’s MR cathepsin-L substrate, MR-(FR)2, contains two pairs of FR coupled to cresyl violet. CTSL activity was measured using the fluorescent activity assay kit according to the manufacturer’s instructions. The fluorescence was monitored by Cytoflex (Beckman Coulter, Brea, CA). It has an optimal excitation of 592 nm and emission of 628 nm. The data were analyzed using FlowJo software (Tree Star, Ashland, OR).

#### Transmission electron microscopy

For transmission electron microscopy examination, HBE cells after experimental manipulations were fixed in 2.5% glutaraldehyde at 4°C overnight. Then the samples were prepared according to standard methods. Images were scanned with TECNA1 10 transmission electron microscope (FEI, Hillsboro, Oregon, USA) at the Center of Cyro-Electron microscopy, Zhejiang University.

### Quantification and statistical analysis

Data were presented as means ± standard error of the mean (SEM). Statistical analysis was performed with GraphPad Prism 8.0 software (GraphPad Software, San Diego, California, USA). Differences between the two groups were identified using Student’s t test and multiple groups using one-way analysis of variance (ANOVA). Correlations were analyzed using Spearman’s correlation analysis. For analysis of the survival rate, the log rank (Mantel-Cox) test was performed. Values of *p* less than 0.05 was considered statistically significant. Detailed information is provided in the figure legends.

## Supplemental information

Document S1. Figures S1–S7, Tables S1–S3, and Data S1

Data S2. The full LC-MS/MS results, related to Figure 2 Excel file containing even more data too large to fit in a PDF.
