# Supplementary material for: Inhibition of cathepsin L ameliorates inflammation through the A20/NF-κB pathway in endotoxin-induced acute lung injury
Source: iScience. 2024 Oct 4;27(11):111024. doi: 10.1016/j.isci.2024.111024 (PMC11570319; doi:10.1016/j.isci.2024.111024)

iScience, Volume *27*

Supplemental information

Inhibition of cathepsin L ameliorates inflammation through the A20/NF-κB pathway in endotoxin-induced acute lung injury

Shiyi Yang, Kaijun Chen, Jinkang Yu, Zhangchu Jin, Min Zhang, Zhouyang Li, Yang Yu, Nanxia Xuan, Baoping Tian, Na Li, Zhengtong Mao, Wenbing Wang, Tianpeng Chen, Yinfang Wu, Yun Zhao, Min Zhang, Xia Fei, Songmin Ying, Wen Li, Fugui Yan, Xingxian Zhang, Gensheng Zhang, Huahao Shen, and Zhihua Chen

Supplementary figures and their legends

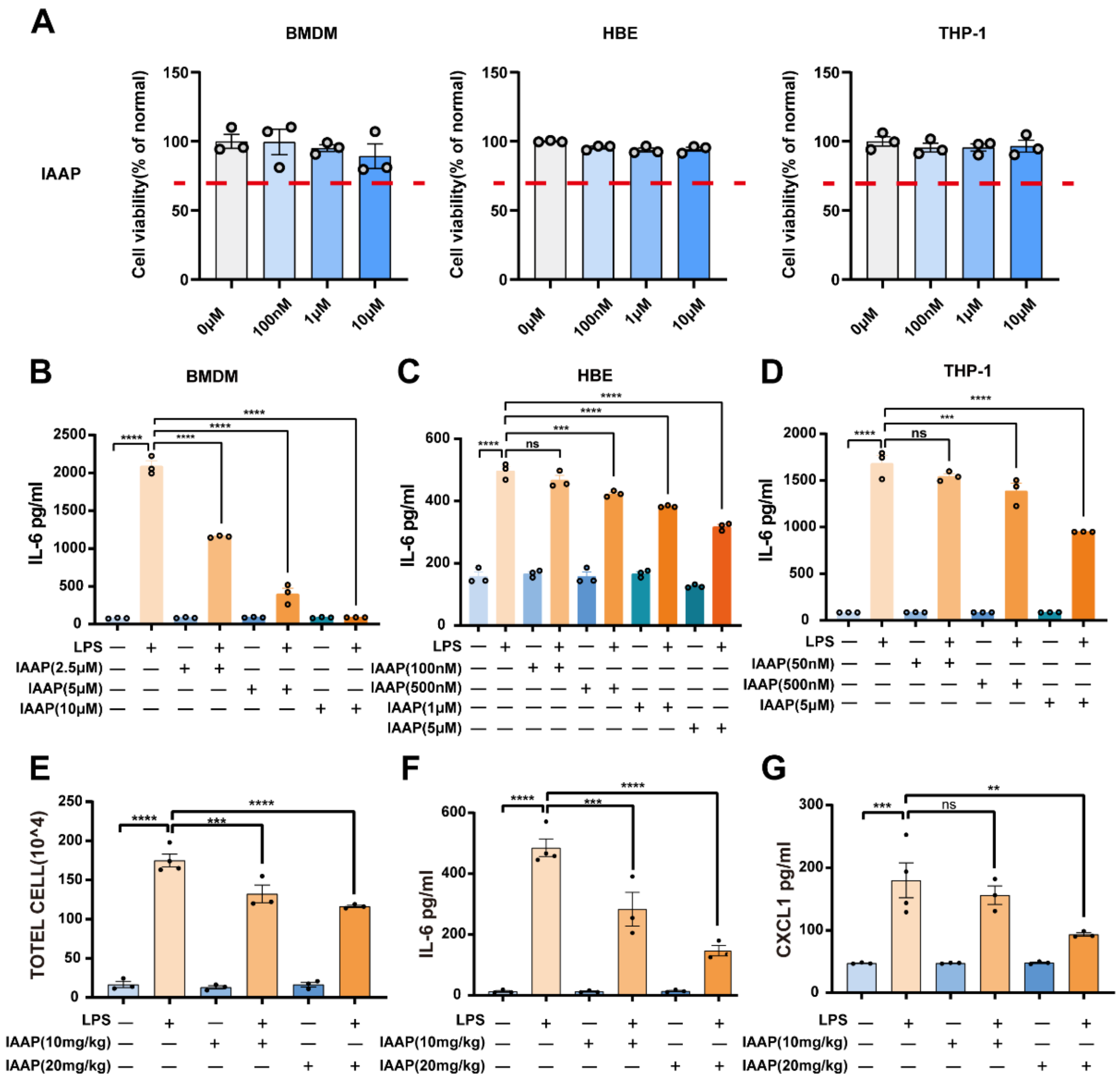

Figure S1. IAAP ameliorates LPS-induced inflammatory response in vitro and ALI in vivo, related to Figure 1.

(A) BMDM and THP-1 were treated with IAAP at different concentrations for 6 h, and HBE were treated with IAAP at different concentrations for 24 h. The cytotoxicity of IAAP in BMDM, HBE, and THP-1 cells were evaluated by MTT assays, n = 3 each group.

(B) BMDM were stimulated with LPS (100 ng/mL), or treated with IAAP at different concentrations for 6 h. Cells were then harvested to analyze secreted IL-6 protein in cell culture supernatants though ELISA, n = 3 each group.

(C) HBE were stimulated with LPS (100 μg/mL), or treated with IAAP at different

concentrations for 24 h. Cells were then harvested to analyze secreted IL-6 protein in cell culture supernatants though ELISA, n = 3 each group.

(D) THP-1 were stimulated with LPS (100 ng/mL), or treated with IAAP at different concentrations for 6 h. Cells were then harvested to analyze secreted IL-6 protein in cell culture supernatants though ELISA, n = 3 each group.

(E) Total cell counts in BALF from mice, n (NS + DMSO) =3, n (LPS + DMSO) =4, n (IAAP (10mg/kg)) =3, n (IAAP (10mg/kg) + LPS) =3, n (IAAP (20mg/kg)) =3, n (IAAP (20mg/kg) + LPS) =3.

(F) Expression of IL6 in the BALF.

(G) Expression of CXCL1 in the BALF.

DMSO was used as the vehicle control. All the quantitative data are presented as mean ± SEM and differences were identified using one-way ANOVA. ***p* <0.01, ****p* <0.001, *****p* <0.0001; ns, *p*> 0.05.

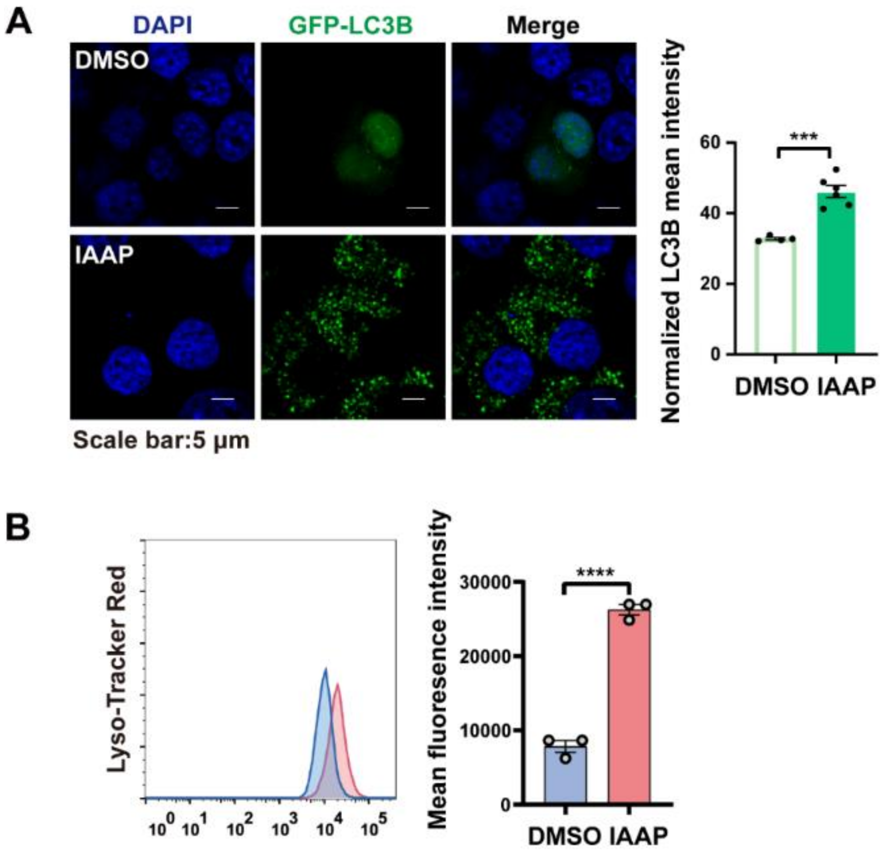

Figure S2. IAAP induces LC3B punctuation and lysosome accumulation by suppressing CTSL, related to Figure 2.

(A) HBE cells stably expressing GFP-LC3B were treated with IAAP (10 μM) for 6 h and were imaged by confocal microscopy. Scale bars: 5 μm. The quantification shown on the right represents the fluorescence intensity of LC3B, n=5.

(B) HBE cells were treated with IAAP at 10 μM for 6 h, then stained with Lyso-Tracker. The Lyso-Tracker fluorescence intensity was detected by flow cytometry. A representative flow cytometry profile is shown on the left. Quantification shown on the right represents the fluorescence intensity of the Lyso-Tracker, n=3.

DMSO serves as the vehicle control. All the quantitative data are presented as mean ± SEM. Differences between the two groups were identified using Student's t-test. ***p <0.001, ****p <0.0001.

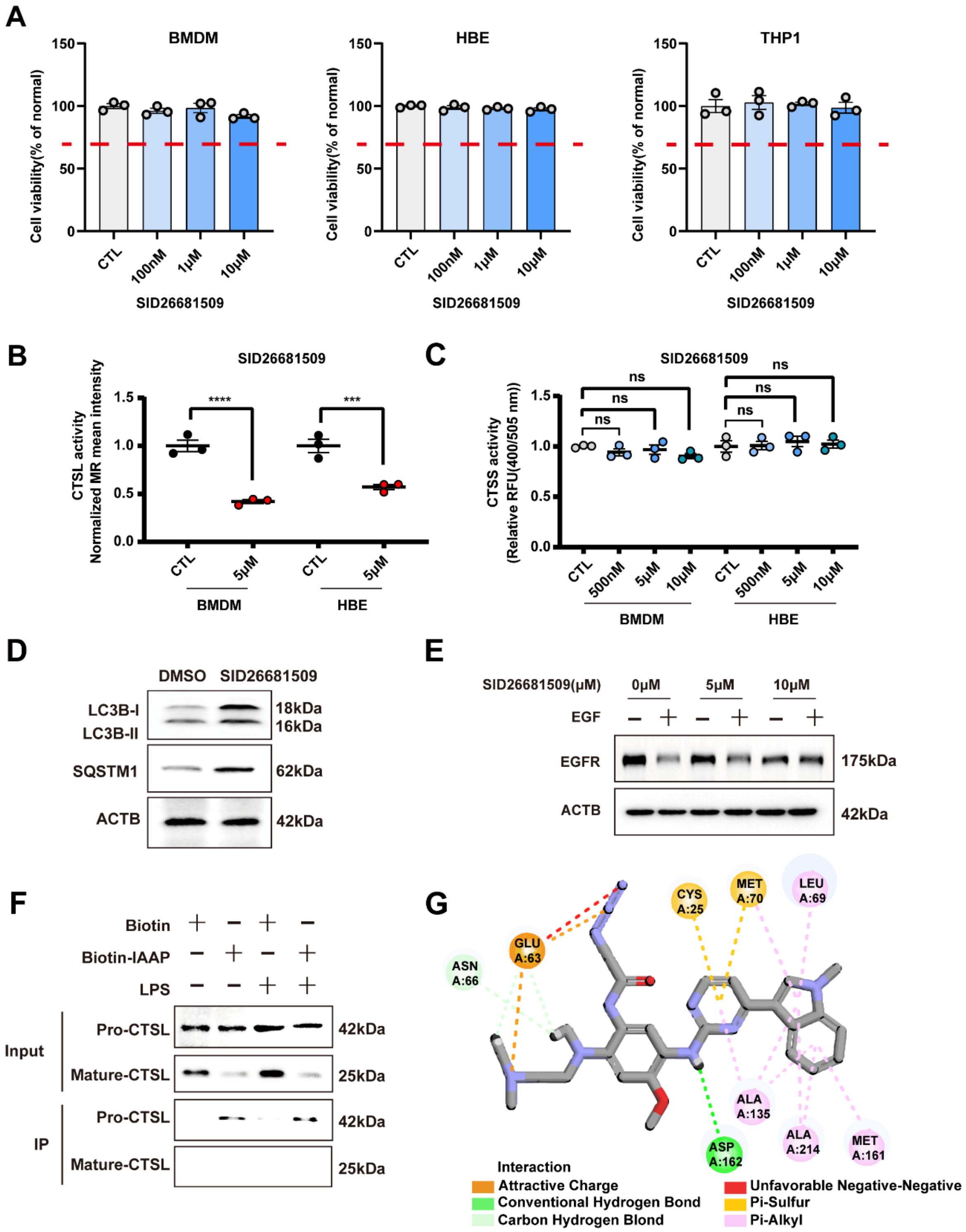

Figure S3. IAAP interacts with CTSL to suppress their bioactivity, of which CTSL is critical for lysosomal function, related to Figure 3.

(A) BMDM and THP-1 were treated with SID26681509 at different concentrations for 6 h, and HBE were treated with SID26681509 at different concentrations for 24 h. The cytotoxicity of SID26681509 in BMDM, HBE, and THP-1 cells were evaluated by MTT assays, n = 3 each group.

(B) BMDM were treated with SID26681509 (5 μM) for 6 h, and HBE were treated with SID26681509 (5 μM) for 24 h. The CTSL activity in BMDM and HBE were evaluated, n = 3 each group.

(C) BMDM were treated with SID26681509 at different concentrations for 6 h, and HBE were treated with SID26681509 at different concentrations for 24 h. The CTSS activity in BMDM and HBE were evaluated, n = 3 each group.

(D) HBE were treated with SID26681509 (5 μM) for 6 h, the levels of LC3B and SQSTM1 were examined using western blotting.

(E) Western blot analysis of EGFR after EGF incubation in HBE cells with SID26681509 (5 μM) treatment for 6 h.

(F) HBE cells were treated with 10 μM biotin or IAAP-biotin respectively, and stimulated with or without LPS. The cell lysates were immunoprecipitated with beads-streptavidin and subjected to immunoblotting.

(G) The planar interaction diagram of IAAP with Cathepsin L shows the molecular surface of the protein and the locations of key residues.

DMSO serves as the vehicle control. All the quantitative data are presented as mean ± SEM. Differences between the two groups were identified using the student's t-test and multiple groups using one-way ANOVA. ***p <0.001, ****p < 0.0001; ns, p > 0.05.

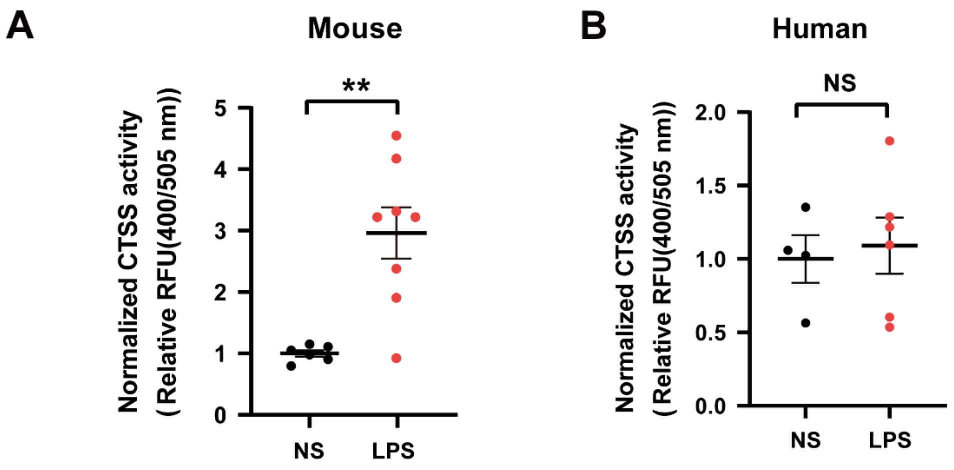

Figure S4. CTSS is elevated in the murine model of LPS-induced ALI, related to Figure 4.

(A) CTSS activity in BALF from from mice, n (NS)=6, n (LPS)=8.

(B) CTSS activity in BALF from healthy controls (n=4) and patients with ARDS (n=6). DMSO serves as the vehicle control. All the quantitative data are presented as mean ± SEM and differences between the two groups were identified using Student's t-test. $^{**}p < 0.01$, ns, $p > 0.05$.

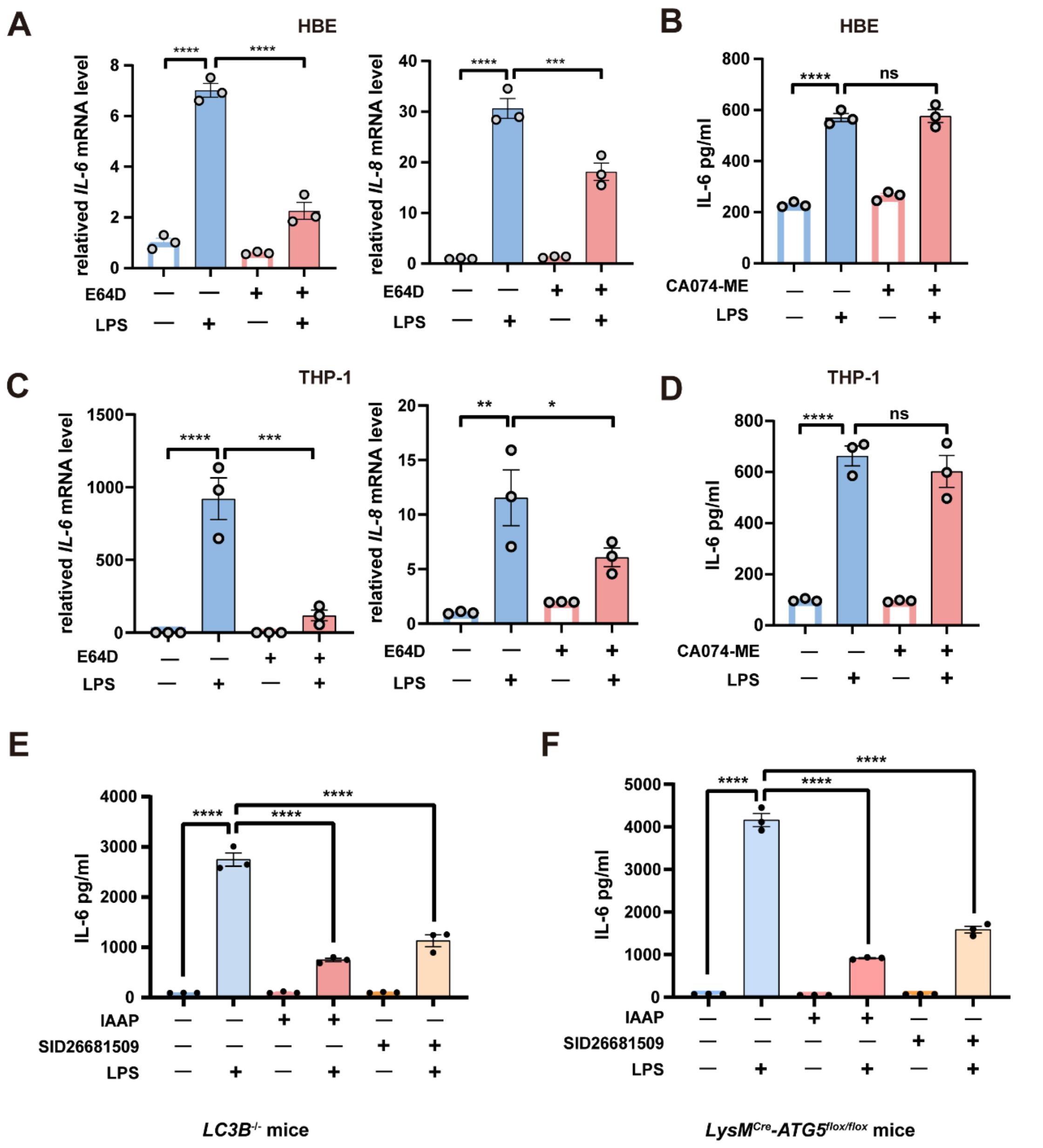

Figure S5. CTSL inhibitors attenuate LPS-induced inflammation independently of autophagy, related to Figure 5.

(A) HBE were stimulated with LPS (100 μg/mL), or treated with E64D (10 μM) for 24 h. Cells were then harvested for analyzing the mRNA expression of IL-6 and IL-8 by Q-PCR, n = 3 each group.

(B) HBE were stimulated with LPS (100 μg/mL), or treated with CA074-ME (10 μM) for 24 h. Cells were then harvested for analyzing secreted protein in cell culture supernatants of IL-6 by ELISA, n = 3 each group.

(C) THP-1 were stimulated with LPS (100 ng/mL), or treated with E64D (10 μM) for 6 h. Cells were then harvested for analyzing the mRNA expression of IL-6 and IL-8 by

Q-PCR, n = 3 each group.

(D) THP-1 were stimulated with LPS (100 ng/mL), or treated with CA074-ME (10 μM) for 6 h. Cells were then harvested for analyzing secreted protein in cell culture supernatants of IL-6 by ELISA, n = 3 each group.

(E) BMDM from LC3B-/- mice were stimulated with LPS (100 ng/mL), or treated with IAAP (5 μM) or SID26681509 (5 μM) for 6 h. Cells were then harvested for analyzing secreted protein in cell culture supernatants of IL-6 by ELISA, n = 3 each group.

(F) BMDM from $LysM^{Cre}$-$Atg5^{flox/flox}$ mice were cotreated with LPS (100 μg/mL), IAAP (5 μM) or SID26681509 (5 μM) for 24 h. Cells were then harvested for analyzing secreted protein in cell culture supernatants of IL-6 by ELISA, n = 3 each group.

DMSO serves as the vehicle control. All the quantitative data are presented as mean ± SEM and differences were identified using one-way ANOVA. *p <0.05, **p < 0.01, ***p < 0.001, ****p < 0.0001, ns; p >0.05.

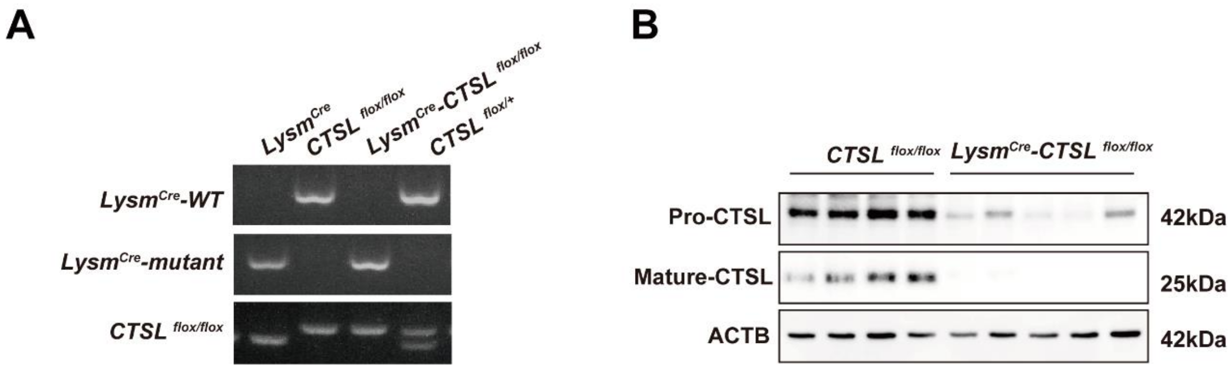

Figure S6. The cell-specific knockdown effects of CTSL in mouse lung tissues, related to Figure 6.

(A) Genotyping was analyzed by PCR using genomic DNA from mouse tails.

(B) Representative immunoblots of CTSL in mouse lung tissues.

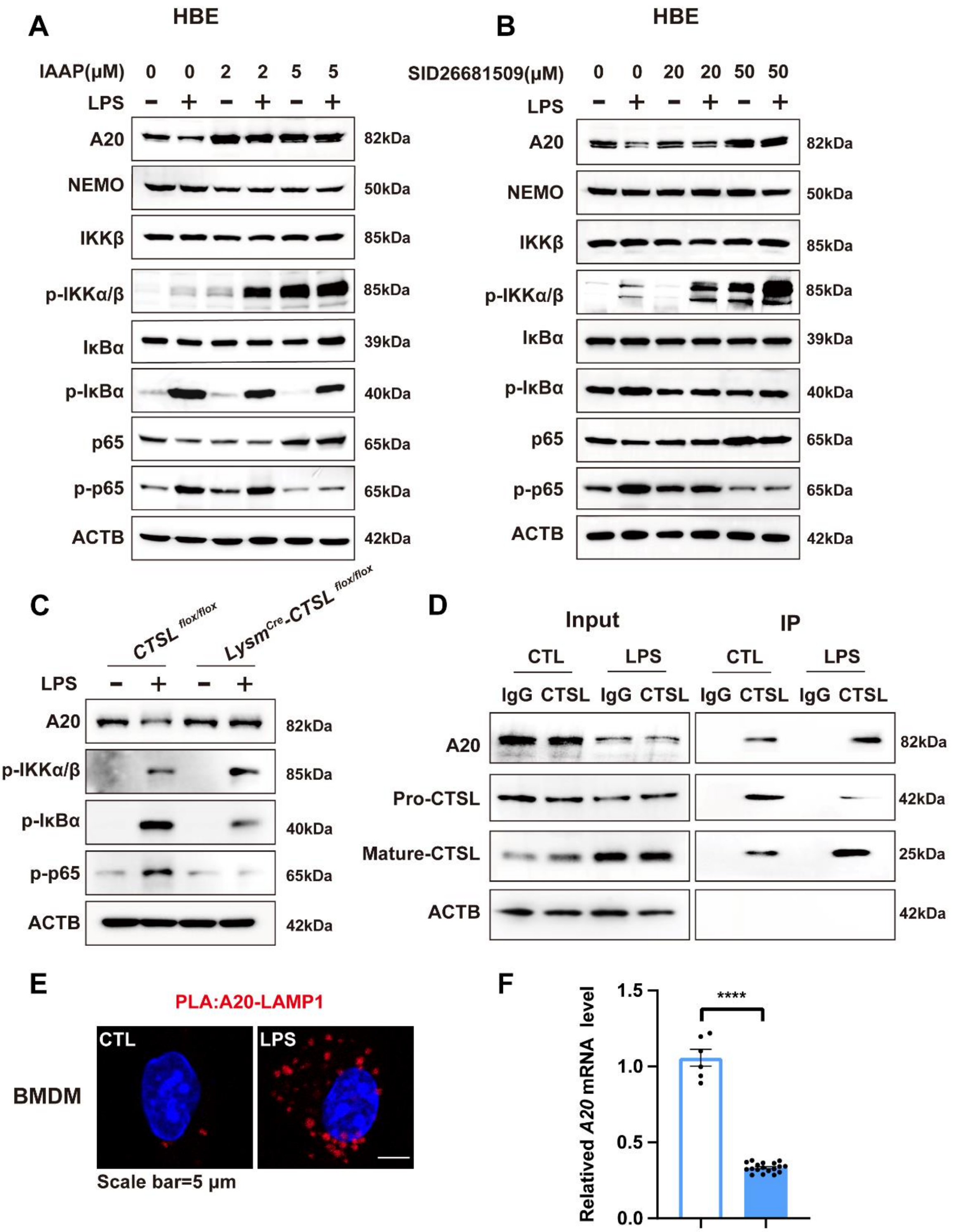

Figure S7. CTSL regulates the NF-κB pathway by degrading A20, related to Figure 7.

(A) HBE cells were treated with IAAP for 6 h, then stimulated with LPS (100 μg/mL) for 20min, the levels of A20, NEMO, IKKβ, p-IKKα/β, IκBα, p-IκBα, p65, p-p65 and ACTB were examined using western blotting.

(B) HBE cells were treated with SID26681509 for 6 h, then stimulated with LPS (100 μg/mL) for 20min, the levels of A20, NEMO, IKKβ, p-IKKα/β, IκBα, p-IκBα, p65, p-p65 and ACTB were examined using western blotting.
(C) BMDM cells were obtained from indicated mice and stimulated with LPS (100 ng/mL) for 5 min. the levels of A20, p-IKKα/β, p-IκBα, p-p65 and ACTB were analyzed using western blotting.
(D) HBE were stimulated with LPS (100 μg/mL) for 20min, the cell lysates were immunoprecipitated with anti-CTSL and subjected to immunoblotting.
(E) BMDM was analyzed for the spatial approximation of A20 with LAMP1 components by PLA. Red, proximity ligation-positive signals. Scale bars, 5μm.
(F) Knockdown efficiency of *A20*-siRNA, n (Control-siRNA) = 6 each group, n (*A20*-siRNA) = 18 each group.
DMSO serves as the vehicle control. All the quantitative data are presented as mean ± SEM. Differences between the two groups were identified using the student t-test. ****$p < 0.0001$.

Supplementary tables

Table S1. Brief information of Healthy Controls and patients with ARDS, related to Figure 4.

	Healthy Controls	ARDS	P-Value (Healthy Controls vs. ARDS)
**Number**	4	6	-
**Age, yrs**	69.25±8.14	78.33±8.69	0.1758
**Sex (M/F)**	2/2	6/0	-
**Sepsis – no.**	0	1	-

Data are presented as means ± SD. Categorical variables in patients' information were analyzed by Pearson's chi square tests where $P < 0.05$ is considered significant.

Table S2. Brief information of ARDS patients, related to Figure 4.

No.	Sex	Age, yrs	IL-6(pg/ml)	Primary diagnosis
1	M	71	48.1	Severe pneumonia
2	M	95	41.4	Severe pneumonia
3	M	77	16.4	Severe pneumonia
4	M	83	218.4	COVID-19 with respiratory failure
5	M	69	77.7	Pulmonary infection
6	M	75	89.0	Acute respiratory failure combined with acute exacerbation of COPD

Table S3. Primers used for quantitative real time PCR analysis, related to Figure 5 and 7.

Human

Gene	Sequences (5' to 3')
IL-6	Forward: ACTCACCTCTTCAGAACGAATTG
IL-6	Reverse: CCATCTTTGGAAGGTTCAGGTTG
IL-8	Forward: ACTGAGAGTGATTGAGAGTGGAC
IL-8	Reverse: AACCCTCTGCACCCAGTTTTC
CTSB	Forward: GCTTCGATGCACGGGAACAATG
CTSB	Reverse: CATTGGTGTGGATGCAGATCCG
CTSL	Forward: ACTGCACAATCAGGAATACAGG
CTSL	Reverse: AAAAGCCCAACAAGAA
β-Actin	Forward: CATGTACGTTGCTATCCAGGC
β-Actin	Reverse: CTCCTTAATGTCACGCACGAT

Mouse

Gene	Sequences (5' to 3')
IL-6	Forward: CTGCAAGAGACTTCCATCCAG
IL-6	Reverse: AGTGGTATAGACAGGTCTGTTGG
CXCL1	Forward: ACCCAAACCGAAGTCATA
CXCL1	Reverse: GGTGCCATCAGAGCAGT
A20	Forward: GGAUCAUCUAUCACUUUAATT
A20	Reverse: UUAAAGUGAUAGAUGAUCCTT
β-Actin	Forward: GGCTGTATTCCCCTCCATCG
β-Actin	Reverse: CCAGTTGGTAACAATGCCATGT

Data S1. Original figures

Related to Figure 3C

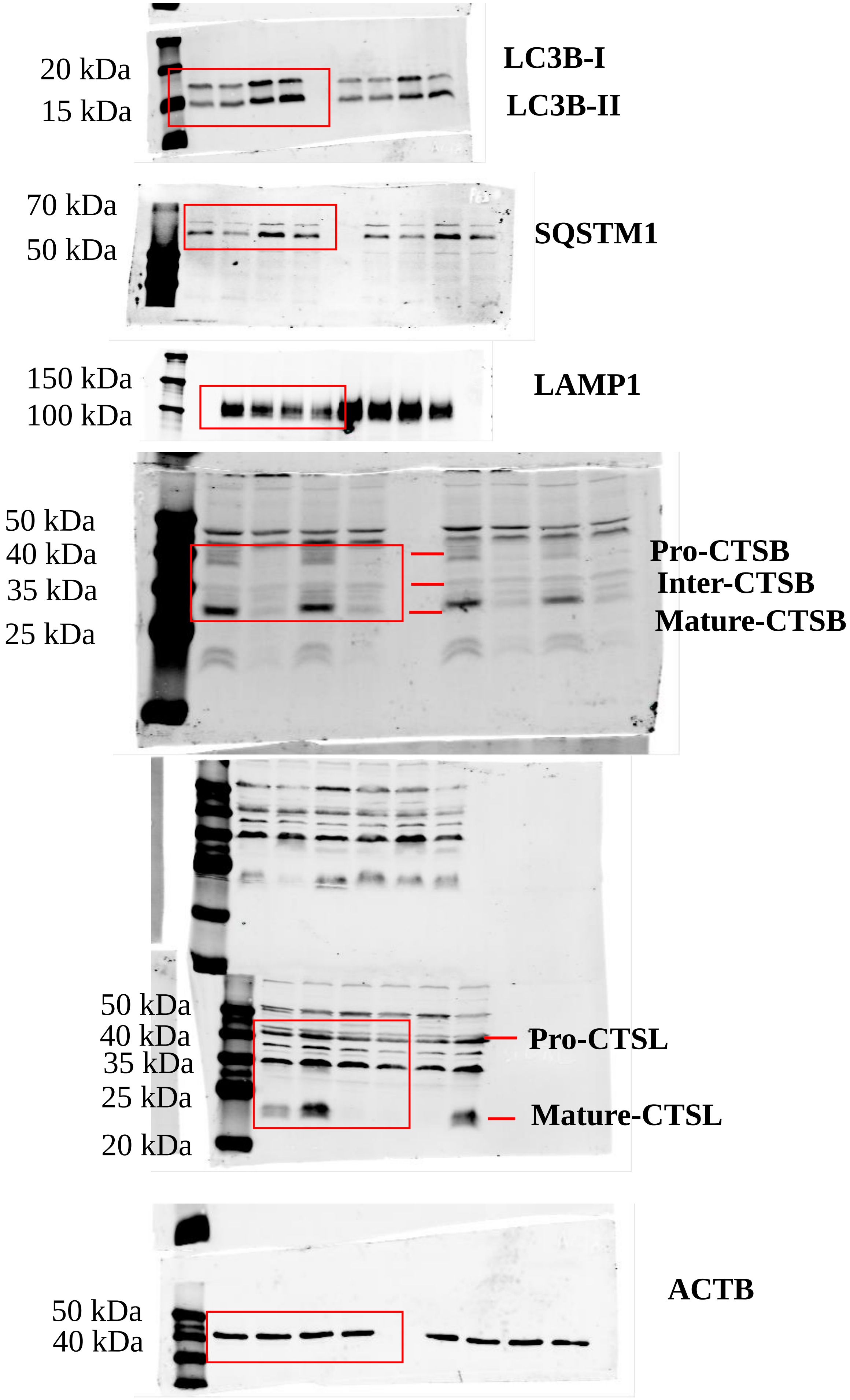

Related to Figure 4F

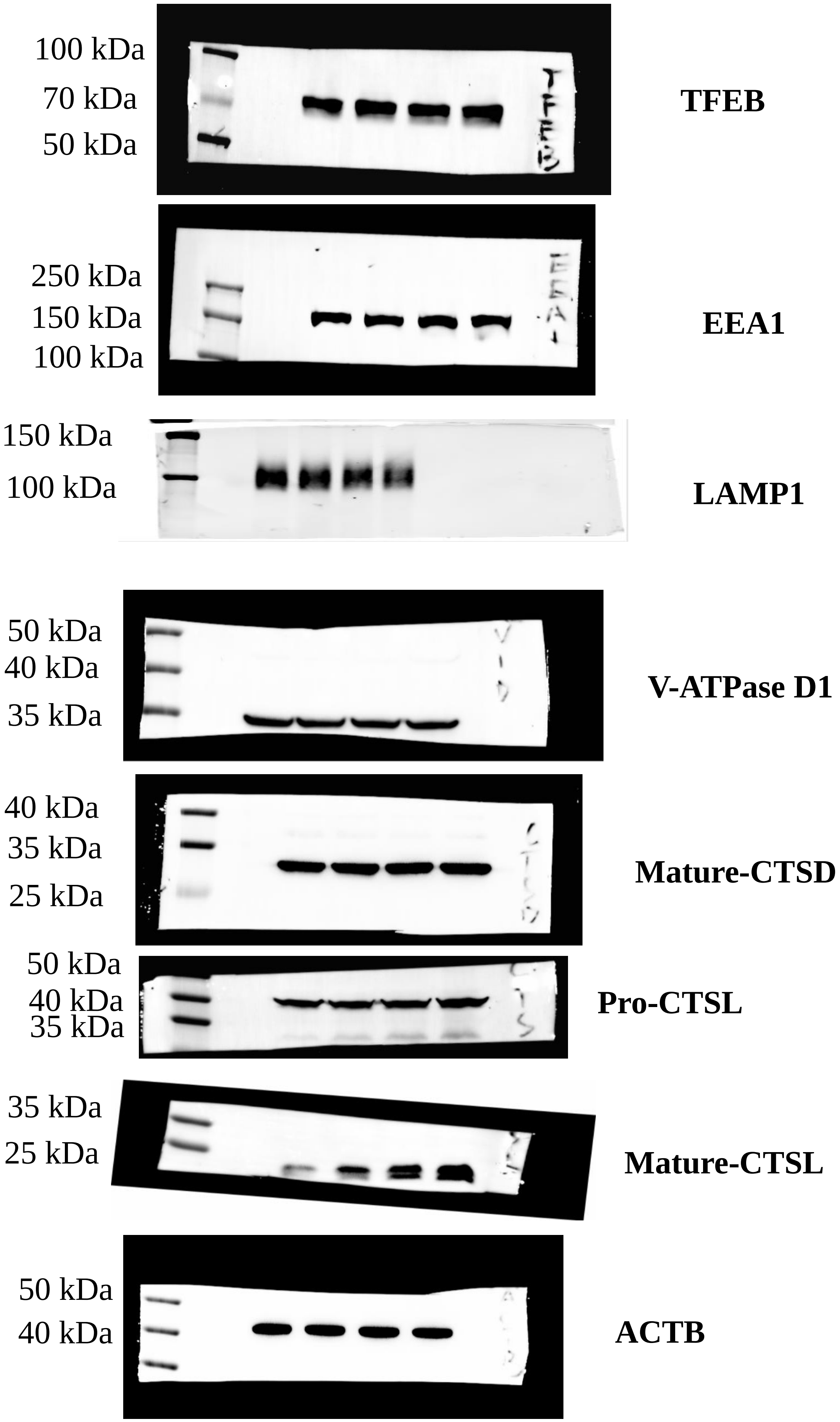

Related to Figure 4G

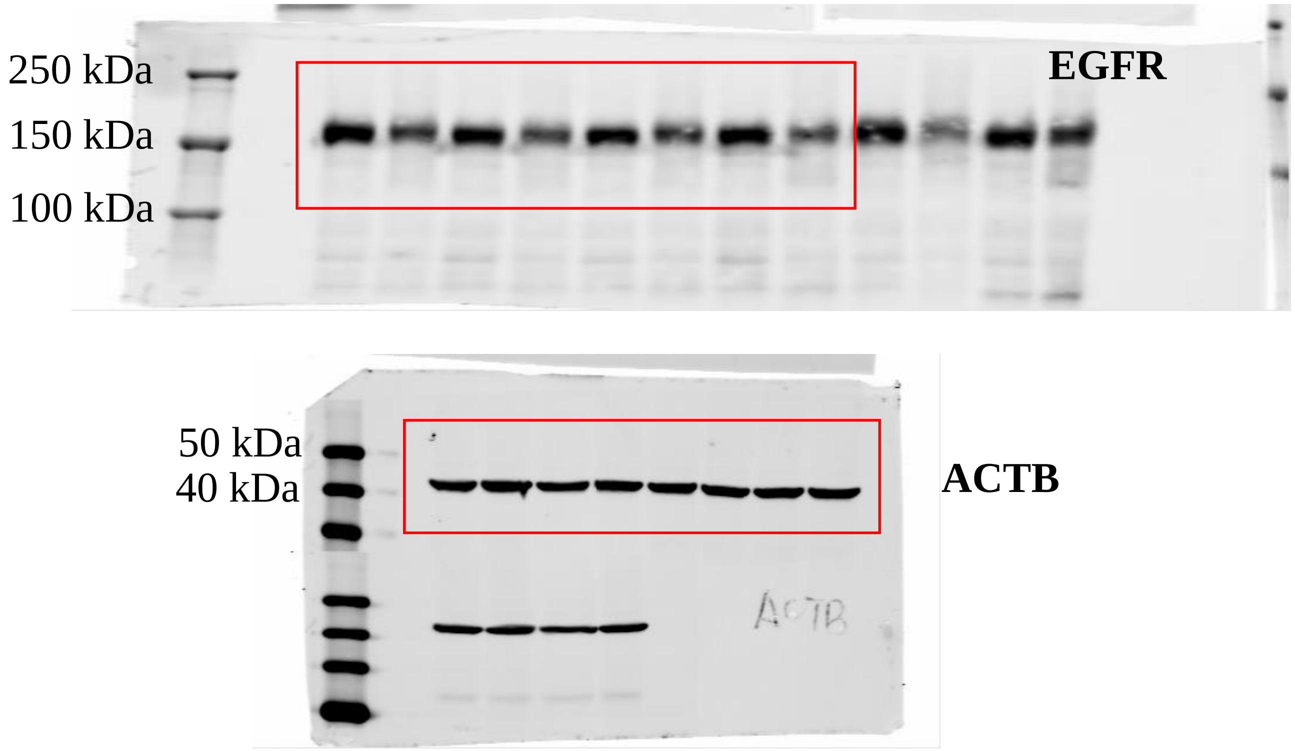

Related to Figure S3E

EGFR

250 kDa
150 kDa
100 kDa

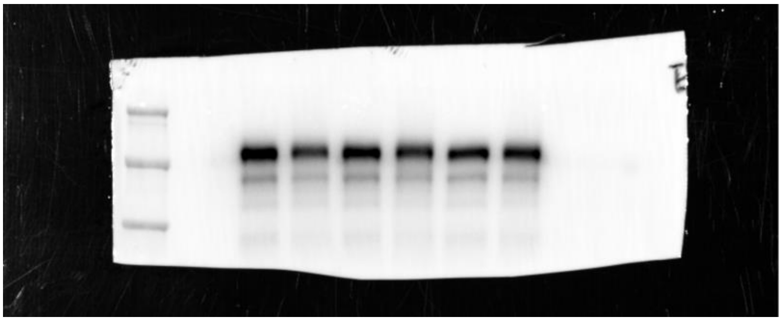

ACTB

50 kDa
40 kDa

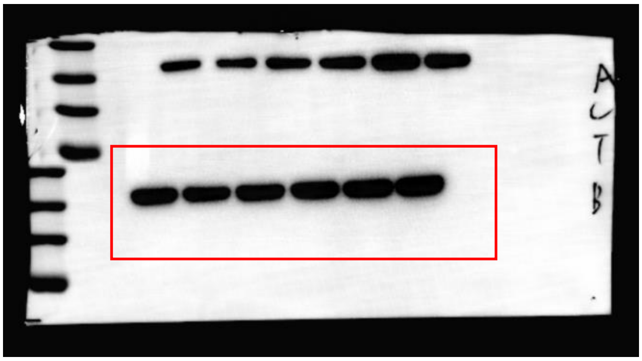

Related to Figure S3G

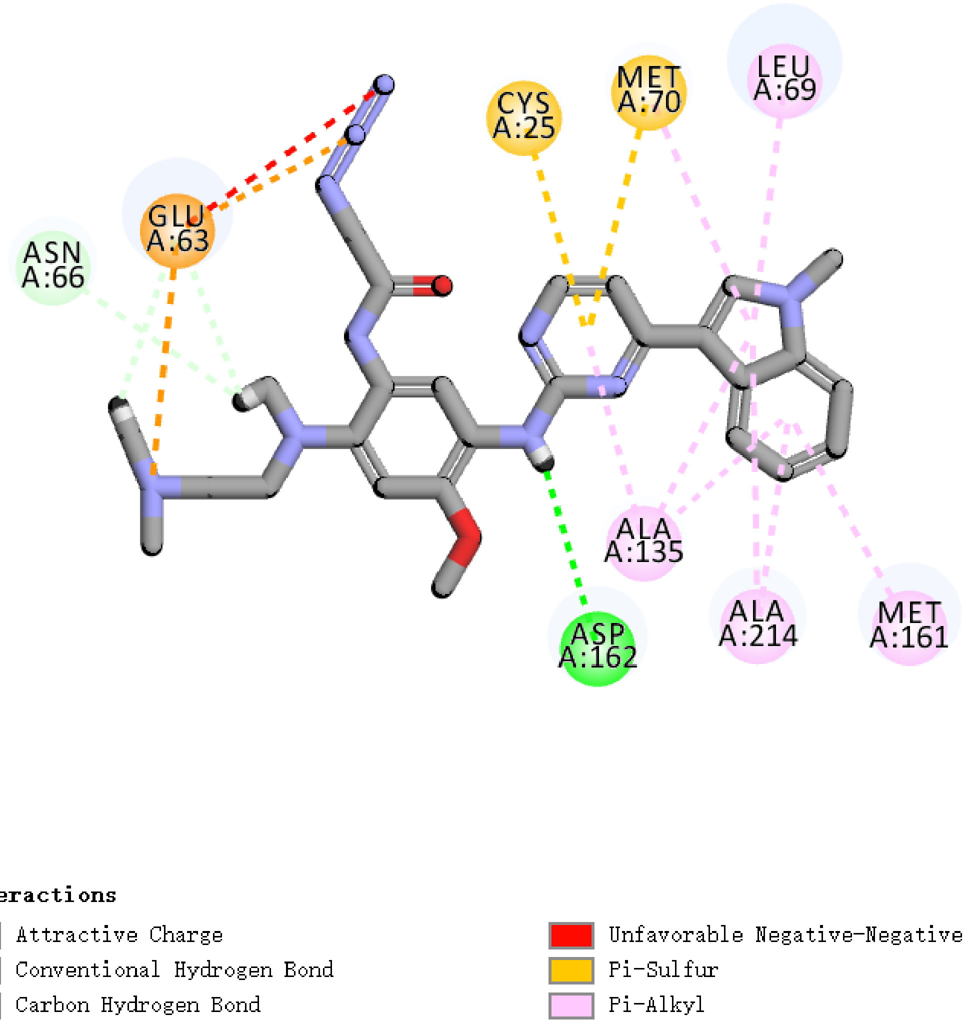

Supplement: Document S1. Figures S1–S7, Tables S1–S3, and Data S1 [file mmc1.pdf]
